# Characterisation of urban environment and activity across space and time using street images and deep learning in Accra

**DOI:** 10.1038/s41598-022-24474-1

**Published:** 2022-11-28

**Authors:** Ricky Nathvani, Sierra N. Clark, Emily Muller, Abosede S. Alli, James E. Bennett, James Nimo, Josephine Bedford Moses, Solomon Baah, A. Barbara Metzler, Michael Brauer, Esra Suel, Allison F. Hughes, Theo Rashid, Emily Gemmell, Simon Moulds, Jill Baumgartner, Mireille Toledano, Ernest Agyemang, George Owusu, Samuel Agyei-Mensah, Raphael E. Arku, Majid Ezzati

**Affiliations:** 1 Department of Epidemiology and Biostatistics, School of Public Health, Imperial College London, London, UK; 2 MRC Centre for Environment and Health, School of Public Health, Imperial College London, London, UK; 3 Department of Environmental Health Sciences, School of Public Health and Health Sciences, University of Massachusetts, Amherst, USA; 4 Department of Physics, University of Ghana, Accra, Ghana; 5 School of Population and Public Health, University of British Columbia, Vancouver, Canada; 6 ETH Zurich, Zurich, Switzerland; 7 Department of Civil and Environmental Engineering, Imperial College London, London, UK; 8 Department of Equity, Ethics and Policy, School of Population and Global Health, McGill University, Montreal, Canada; 9 Department of Epidemiology and Biostatistics, School of Population and Global Health, McGill University, Montreal, Canada; 10 Mohn Centre for Children’s Health and Wellbeing, School of Public Health, Imperial College London, London, UK; 11 Department of Geography and Resource Development, University of Ghana, Accra, Ghana; 12 Institute of Statistical, Social and Economic Research, University of Ghana, Accra, Ghana; 13 Regional Institute for Population Studies, University of Ghana, Accra, Ghana

**Keywords:** Environmental impact, Sustainability

## Abstract

The urban environment influences human health, safety and wellbeing. Cities in Africa are growing faster than other regions but have limited data to guide urban planning and policies. Our aim was to use smart sensing and analytics to characterise the spatial patterns and temporal dynamics of features of the urban environment relevant for health, liveability, safety and sustainability. We collected a novel dataset of 2.1 million time-lapsed day and night images at 145 representative locations throughout the Metropolis of Accra, Ghana. We manually labelled a subset of 1,250 images for 20 contextually relevant objects and used transfer learning with data augmentation to retrain a convolutional neural network to detect them in the remaining images. We identified 23.5 million instances of these objects including 9.66 million instances of persons (41% of all objects), followed by cars (4.19 million, 18%), umbrellas (3.00 million, 13%), and informally operated minibuses known as tro tros (2.94 million, 13%). People, large vehicles and market-related objects were most common in the commercial core and densely populated informal neighbourhoods, while refuse and animals were most observed in the peripheries. The daily variability of objects was smallest in densely populated settlements and largest in the commercial centre. Our novel data and methodology shows that smart sensing and analytics can inform planning and policy decisions for making cities more liveable, equitable, sustainable and healthy.

## Introduction

The environments in which the inhabitants of cities live, work and travel can influence their health, safety and wellbeing both positively and adversely^1^. For instance, different modes of transportation, such as walking, cycling, driving private cars or motorcycles, and taking public transportation, have implications for ease and cost of mobility and are associated with different risks of injury, levels of physical activity, and emissions of, and exposures to, air and noise pollution^2^. City markets provide a setting for income generation, access to goods and services and social interactions but also can be settings of social conflict and confrontations^3–6^. Single-use plastics, which are a common form of packaging found in many cities, enable working people to have ready access to food, beverage, and goods but are important contributors to solid waste which can block drainage channels (e.g., gutters) if their disposal is not properly managed^7^. This can lead to water logging problems and exacerbate urban flood risk^7,8^, which often disproportionately impacts poor communities^9,10^. Build-up of trash^9^ and the free roaming of livestock and other animals^11,12^ can increase the population and diversity of disease vectors. Some features of city environments that affect health, safety and wellbeing are stationary over short periods of time (e.g., buildings, trees and other forms of greenspace). Others, such as traffic or market activities, vary across space and time, as does the scale at which people carry out their daily activities. The combination of how people spend their time in and travel through different parts of a city, and the dynamics of environmental features and the objects that influence them, patterns a population’s experience of the city’s social, commercial and built environments.

The number of people living in cities in sub-Saharan Africa (SSA) increased from 51 million in 1970 to 450 million in 2020, growing faster than any other region^13^. As cities across SSA grow and change, there is a critical need to understand the spatial and temporal dynamics of urban environmental features and human–environment interactions that are relevant for health, safety and wellbeing. This information is essential for the formulation and evaluation of policies that promote positive outcomes, especially as such policies may be different from those in industrialised countries^1,14,15^. Our aim was to characterise spatial patterns and temporal dynamics of features of the urban environment that are relevant for health, safety and sustainability, across different neighbourhood types and time scales within a major SSA city, and to provide an approach for doing so in other cities in Africa and throughout other low and middle-income countries. To achieve this aim we collected a novel and bespoke image dataset in the city of Accra, Ghana, and adapted and applied a convolutional neural network (CNN) to these images, to detect features which help to understand the patterns and dynamics of human activity and environment.

## Data, methodological context and contributions

Censuses, other routinely collected government data, and economic, transport and health surveys have demonstrated that African cities are expanding rapidly, accompanied by changes in population age structure, socioeconomic status, sanitation and transportation infrastructure, and housing characteristics^14,16^. However, census and large survey data are costly, take a long time to plan and implement, typically every few years, and hence lack the temporal resolution needed to understand the dynamics of urban life and environment at shorter timescales. These types of data are usually collected at the level of households or neighbourhoods^17,18^, and do not capture the use of, and interactions with, urban public spaces such as streets, roads and marketplaces. In the sections below, we describe how smart sensing of cities can provide complimentary types of data to censuses and surveys.

Researchers and policy agencies have recently incorporated different kinds of passive and active smart sensing data to study spatially and temporally varying urban environments and activities. The availability, information content, and the spatial and temporal scales of such data vary by domain of urban activity, including transportation, energy, water, telecommunication and retail^19^. The current collection and use of such data is limited in SSA countries^20^. Time-stamped and spatially resolved data from phone network usage, mobile social media or Global Positioning Systems (GPS) have been used to study mobility in cities^21–23^, including in some SSA cities^24–27^, including sudden changes in their dynamics, for example during the first few months of the COVID-19 pandemic and the corresponding social distancing measures^28^. However, information on urban environments is typically not available from mobile phone data^29^. Furthermore, such data, particularly in the SSA context, where there are a large number of urban poor, are not representative of the population since socioeconomic factors influence the ownership, type (e.g. smartphone vs feature phone) and frequency of mobile phone use^30,31^.

Short-term observations in specific locations in some SSA cities have included rich environmental data^32–34^, but are limited in spatial and temporal coverage. Street-level images, if collected with high spatial coverage, provide granular information on the urban environment and its spatial variations^35–41^, which can be extracted using computer vision techniques^37^. However, imagery data from cities in African countries are limited in quantity, with some parts of the city less represented^42^. Further, the temporal resolution of such data, typically on an annual basis and primarily during the day, makes it best suited for the analysis of static or slow changing features or average trends. For example, unlike mobile phone data, street images from sources such as Google Street View, have not been used for evaluating changes in urban activity with respect to COVID-19 lockdowns. The methods used to extract features from images have overwhelmingly relied on CNNs^37^ including object detection methods^43,44^ for countable and interpretable features (namely “objects”), such as persons^45,46^, safety barriers^47^, vehicles^48^ or street signage^49^. Because such algorithms were largely trained using data from high-income countries, they are biased towards representations of objects from these countries, leading to misidentification and poorer accuracy when applied to images elsewhere^31,50^.

Our study makes a number of novel contributions to the use of smart sensing and analytics to understand the urban environment and its impacts on people, especially in rapidly expanding cities in SSA and other developing regions. We present a unique dataset that was created through distributed collection of over two million street-level images at 145 representative sites throughout Accra with high temporal resolution. We systematically adapted computer vision techniques—including transfer learning and data augmentation—to the local environmental and social context. Using these data and methods, the paper presents a characterisation of the dynamics of urban human activity and environment, methodologically bridging the gap between studies focused on spatiotemporal urban mobility patterns and those extracting features of environments from images. We also evaluated whether this approach could detect the impact of policies and interventions on neighbourhood environment, traffic and/or human activities, and how the effects varied across the city. Such a policy was implemented in Accra in response to the COVID-19 pandemic (April 2020 city-wide lockdown), which coincided with our data collection campaign. Finally, we discuss how our results and approach can be used to address data gaps related to the urban environment relevant for health and wellbeing in SSA, and support urban planning and policy decisions to make cities more equitable, sustainable and healthy.

## Study location

Our work covered the Greater Accra Metropolitan Area (GAMA, ~ 1500 km^2^), the administrative boundary of Accra, the capital and largest city in Ghana, with a population of about five million^51^. GAMA comprises the Accra Metropolitan Area (AMA, ~ 2 million people) at its core, other metropolises and municipalities (e.g., Industrial port city of Tema), and peri-urban and largely rural areas in the periphery. Accra has become one of SSA’s leading hubs for business, technology and education^14,52^, with large variations and inequalities in individual and community wealth, and a diversity of ethnicities and languages^14,52^. Accra has diverse land use, built environment, and provision of services which influence the spatial and temporal patterns of health determinants and outcomes^53,54^. There is virtually no train or tram service beyond a shuttle train between Accra central and Tema, and formal transit bus services are limited^52^. Therefore, private vehicles and privately owned and informally operated minibuses, known locally as tro tro are the main means of public transportation^55,56^ along with ride-share cars (e.g. Uber) and motorcycle-taxis.

## Study design

**Figure 1:**
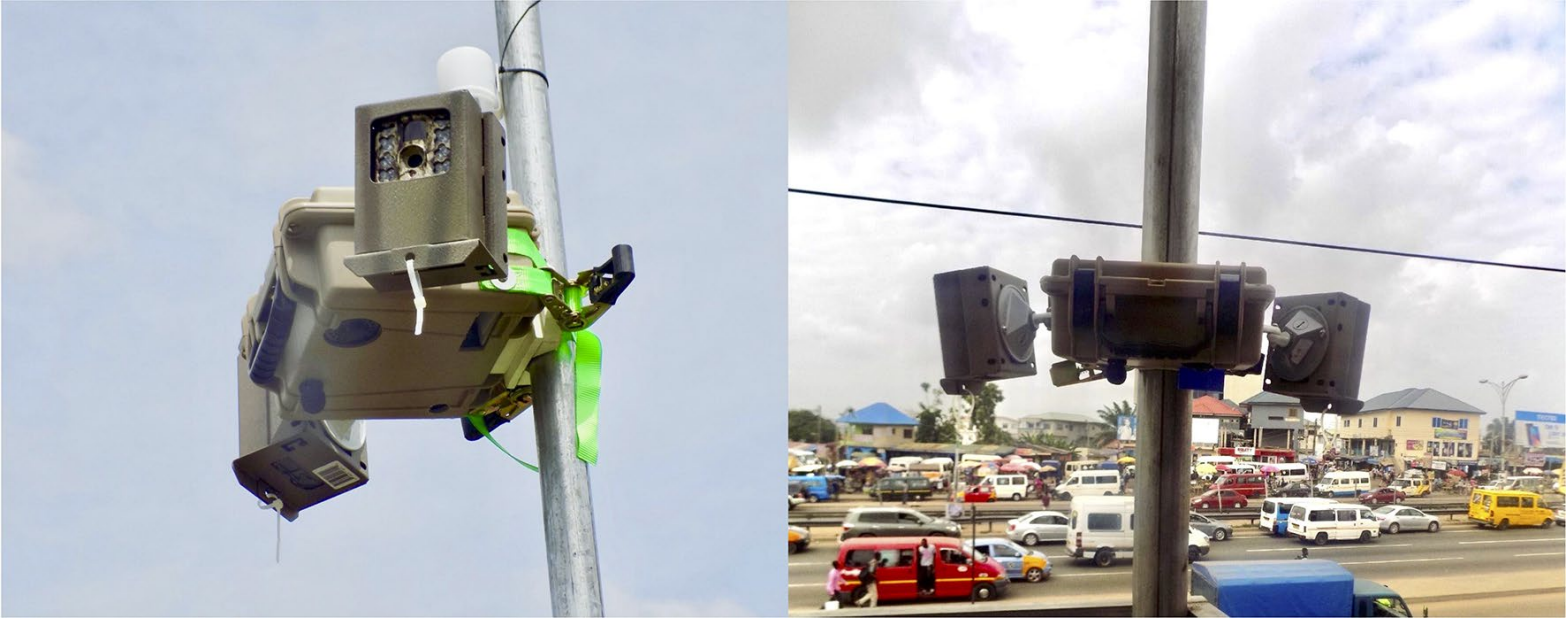
Examples of cameras used for image capture and their installation at measurement sites.

Over a ~ 15 month period, we placed cameras (Fig. 1) at 145 sites throughout the Greater Accra Metropolitan Area (GAMA) for either weeklong (n = 135 sites) or ~ year long periods (n = 10 sites) capturing ~ 2.10 million images^57^. The sites were selected to be representative of the city’s diverse social, physical and natural environment, and were sampled as described in the study protocol paper^57^ from areas classified as i) formal, mostly low- and medium-density, residential areas; ii) informal, mostly high-density, settlements and slums; iii) commercial, business and industrial (CBI) areas; and iv) “other” areas that are often peri-urban or rural, and can have dense vegetation (i.e., forest, grassland) or barren land (i.e., sand, soil, dirt).

**Figure 2:**
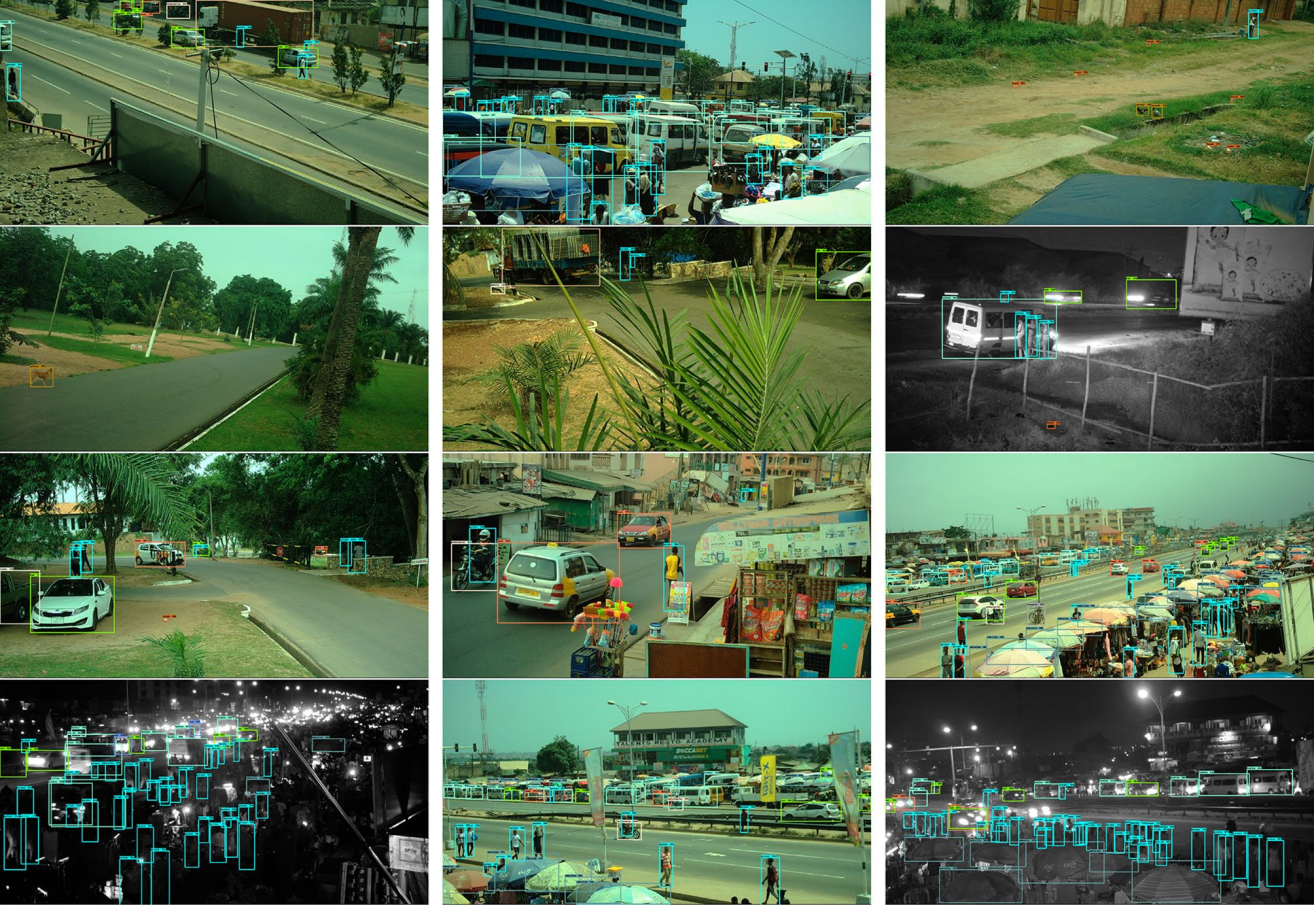
Objects identified in example images. Each identified object is bounded by a box, coloured by object type. The number next to the object’s name shows the final layer’s activation score for the given object’s classifier, which may be heuristically interpreted as the network’s confidence score in its prediction. “Truck” refers to pick-up truck, “Bowl” refers to cooking bowl/pot, “Stall” refers to market stalls and “Stove” to cookstoves of the type found in markets.

We used an interdisciplinary consensus process to identify 20 distinct objects within the images which are relevant for mobility, safety, leisure and play, daily life activities like shopping, air and noise pollution, and sanitation and hygiene. These objects were grouped as persons and market vendors; large vehicles (lorries, vans, buses and tro tros); small vehicles (cars, taxis and pick-up trucks); two wheelers (bicycles and motorcycles); objects from the market and street vending (market stalls, umbrellas, cookstoves, cooking pots/bowls, food and loudspeakers) which are common in African cities^5,6^; refuse (debris and trash); and animals. Members of the research team identified and labelled these objects in bounding boxes across 1,250 images. We then divided the labelled images into training, testing and validation sets and used them to retrain and test the performance of a pre-trained CNN as described in Methods and Data. The retrained CNN was then applied to the entire image set to identify the candidate objects in each image (Fig. 2).

## Results

### Number of people and objects

**Figure 3:**
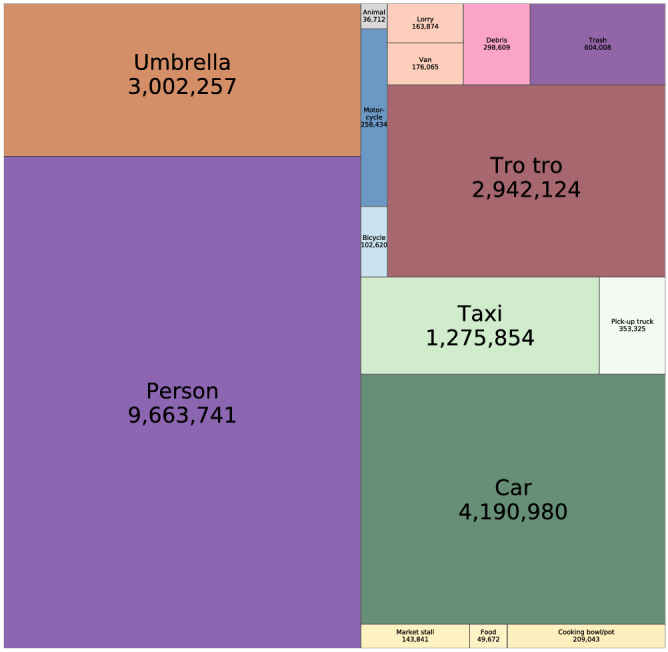
Count of different objects in the Accra image set. The size of each rectangle shows the total numbers of the corresponding object in the entire set of 2.10 million images. Four of the identified objects are not seen in the figure because their numbers were zero (loudspeakers and cookstoves) or very small (98 market vendors and 14 buses).

By applying the algorithm described in Methods and Data, we identified 23.5 million occurrences of the 20 target objects in our 2.10 million images. Of these, 9.66 million (41%) were persons, followed by cars (4.19 million; 18%), umbrellas (3.00 million; 13%) and tro tros (2.94 million; 13%) (Fig. 3). The large number of umbrellas reflect their use over many hours at the same place to protect market and roadside vendors and their merchandise from the sun and rain. The least common objects that were routinely identified were animals (36,712; 0.2%), food (49,672; 0.2%) and bicycles (102,620; 0.4%). Furthermore, our network identified no cookstoves or loudspeakers, and only 14 buses and 98 market vendors.

### Spatial patterns and correlations of people and objects

**Figure 4:**
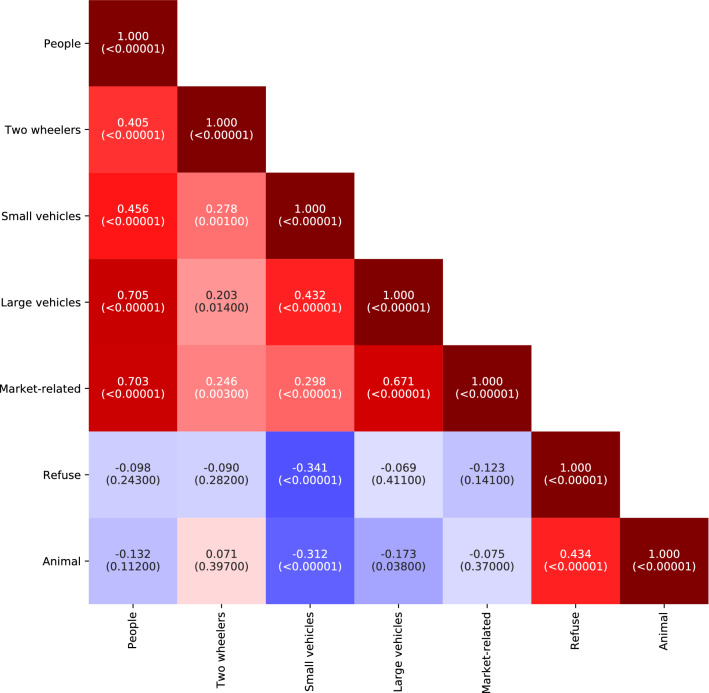
Co-occurrence of objects at measurement sites. The figure shows the Pearson correlation coefficient among pairs of object categories for average counts of objects per site, calculated across all cameras at all sites. For sites with two cameras the correlation coefficient was calculated for the average count across both cameras. The number in parentheses shows the p-value for the correlation coefficient. See Supplementary Fig. 3 for correlation coefficients calculated across images, i.e., co-occurrence in images.

**Figure 5:**
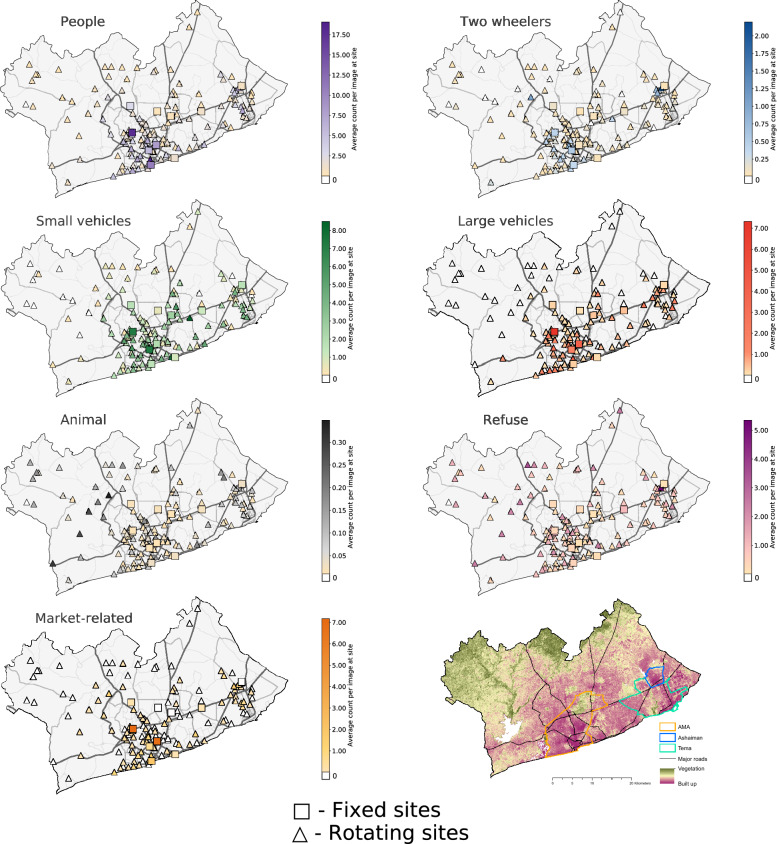
Location of image collection sites and object counts at each site. The 20 objects are categorised into: people (persons and market vendors); large vehicles (lorries, vans, tro tros and buses); small vehicles (cars, taxis and pick-up trucks); two wheelers (bicycles and motorcycles); objects from the market (umbrellas, market stalls, cookstoves, cooking pots/bowls, food and loudspeakers); refuse (trash and debris); and animals. Squares on the maps show the fixed sites and triangles the rotating sites. For each measurement site and object category, the mean number of objects per image is shown. For sites with two cameras, the number shows the mean per image across both cameras. Dark grey lines show major roads and light grey lines secondary and tertiary roads. Road network data are from OpenStreetMap (2019) and the Greater Accra Metropolitan Area (GAMA) boundary, as well as those of Accra Metropolitan Area (AMA), Ashaiman and Tema, from the Ghana Statistical Service. The bottom right panel map shows how much an area is built-up versus those that are abundant with vegetation, as represented by the Normalised Difference Vegetation Index (NDVI) derived from Landsat 8 Satellite images^98^ (January 2020, 30-m resolution).

The average counts of people, vehicles and market-related objects in images was correlated across sites (Fig. 4), though there was a stronger correlation among the number of people, large vehicles and market-related objects (correlation coefficients ranging from 0.67 to 0.71), than among these three categories and small vehicles (0.30–0.46) (Fig. 4). Large vehicles (7.32 average counts per image) and market-related objects (7.17 counts) were the most common at a site on a major throughput road (N1 West, Lapaz) that traverses northern AMA (Fig. 5). This site also had the second (17.90 counts) and fourth (7.30 counts) most common occurrences of people and small vehicles, respectively. The inspection of images and observations by the authors indicate that many people walk along this road or wait for and alight from tro tros that connect major parts of the city, which makes it attractive to roadside vendors.

More generally, small vehicles were most frequently found along secondary (average of 3.57 counts per image) and major (3.74 counts) roads, especially those in the AMA and in the municipality of Tema and were the only object category which outnumbered counts of people along major roads (Table 1). Large vehicles were most commonly found on secondary roads (1.61 counts), nearly five times as many as along tertiary roads (0.34 counts) and twice as many as along major roads (0.79 counts). Both small and large vehicles were more frequent in the centre of GAMA (obtained from the population-weighted average of centroids of census enumeration areas from the 2010 national Ghana census, corresponding to a location three kilometres west of Kotoka International Airport) than in peripheral areas (p-value for distance-from-centre gradient =  < 0.0001 for small vehicles and 0.02 for large vehicles). They were also most prevalent at the CBI sites, 50% greater than in high-density residential sites and twice as frequent as in low- and medium-density residential and peri-urban sites (Table 2). Two wheelers were present five-fold more frequently in high-density residential (0.26 counts) and CBI (0.22) sites than other categories but showed no specific spatial pattern as a function of distance from the centre of Accra (p = 0.74).

**Table 1:** Object counts by the type of road on which sites were located. Mean counts per image for each object category, shown in Fig. 5, were averaged across sites located on the category of road at which the site was located. Road type from OpenStreetMap (2019).

	Tertiary roads	Secondary roads	Major roads
People	1.79	5.10	3.18
Two wheelers	0.11	0.27	0.19
Small vehicles	1.25	3.57	3.74
Large vehicles	0.34	1.61	0.79
Market-related	0.22	1.56	0.29
Refuse	0.83	0.41	0.50
Animals	0.06	0.01	0.02

**Table 2:** Object counts by the type of area on which sites were located. Mean counts per image for each object category, shown in Fig. 5, were averaged across sites by land use categories.

	Low- and medium-density residential	High-density residential	Commercial, business and industrial	Peri-urban and background
People	1.44	4.18	3.55	0.41
Two wheelers	0.07	0.26	0.22	0.03
Small vehicles	1.29	2.24	3.46	0.30
Large vehicles	0.20	0.75	1.29	0.03
Market-related	0.15	0.81	0.57	0.01
Refuse	0.82	0.81	0.46	0.89
Animals	0.06	0.05	0.02	0.06

The presence of market-related objects, like market stalls, umbrellas that shade vendors from the sun, and cooking pots/bowls, largely followed that of people (correlation coefficient = 0.70) (Fig. 4). Within the AMA, people and market-related objects were more frequently observed in high-density residential sites than in CBI ones and nearly three-fold more frequently than in low- and medium-density residential and peri-urban sites (Table 2). Previous studies have indicated that many residents of these neighbourhoods buy food and household items from these vendors, and that in informal neighbourhoods, many families have home-based enterprises and roadside food vending^33,58,59^, which may explain these patterns. Similar to vehicles, counts of both people and market related objects decreased with distance from the centre of GAMA (p = 0.002 and 0.03 respectively).

Animals and refuse were weakly and inversely correlated with other objects across sites but were themselves positively correlated, since both appeared more frequently in the mostly rural and peri-urban peripheral areas of GAMA (p = 0.004 for refuse and < 0.0001 for animals) and were less frequently observed in CBI areas (Table 2).

### Temporal dynamics of people and objects

**Figure 6:**
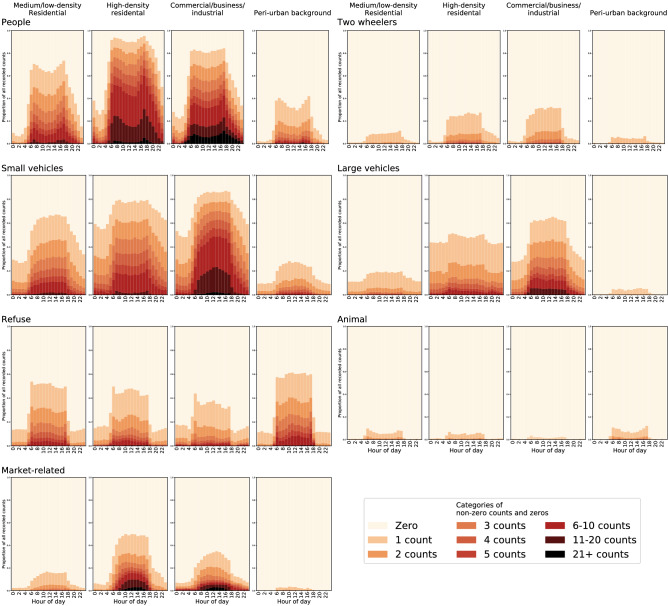
Object count distributions at different hours of the day. Each panel shows the distribution of object counts at each hour of the day for a group of sites. The sites are divided by land-use type: low- and medium-density (formal) residential; informal, mostly high-density, settlements and slums; commercial, business and industrial; and peri-urban areas that are predominantly forest, farmland, grassland or barren land. For this figure, data from the fixed sites, which were operated for the entire year, were down-sampled as described in Methods to produce the equivalent of one week of images, so that each fixed and rotating site contributes the same amount of information. Supplementary Figs. 4 and 5 show the results for data from only fixed sites and only rotating sites, respectively.

The frequency of visible objects changed throughout the day and night times, and the extent of variation depended on both the type of object and site location (Fig. 6). When comparing changes in the proportion of images with one or more counts of a given object across different times of day, presence of people increased sharply between midnight and sunrise (6:00) (604–826% increase at different site types), followed by a midday drop before increasing again at sunset (18:00). At sites in all land use categories, market related objects had a unimodal pattern, peaking at midday, possibly because the sun is at peak intensity, leading to more umbrellas (which shade vendors and their produce) to be visible.

In the high-density informal settlements, people were visible in 30–60% of images at different hours during the night, contrasting with peri-urban and low- and medium-density residential areas, where the majority of night-time images contained no people (Fig. 6). Nonetheless, even in high-density settlements, the number of images with six or more people decreased to virtually zero at night. In all but peri-urban areas, small and large vehicles were present in 20–85% of day-time (6:00–18:00) images but only 10–60% of night-time (18:00–6:00) images (Fig. 6). The night-time decline in the proportion of images with one or more counts of vehicles relative to sunset (18:00) was smallest (5–21%) in high-density sites. Manual observation of the images indicated that this relative stability arose from a combination of continued activity at night-time and roadside vehicle parking. Very few (< 5%) night-time images were found to contain animals (Fig. 6).

### COVID-19 analysis

**Figure 7:**
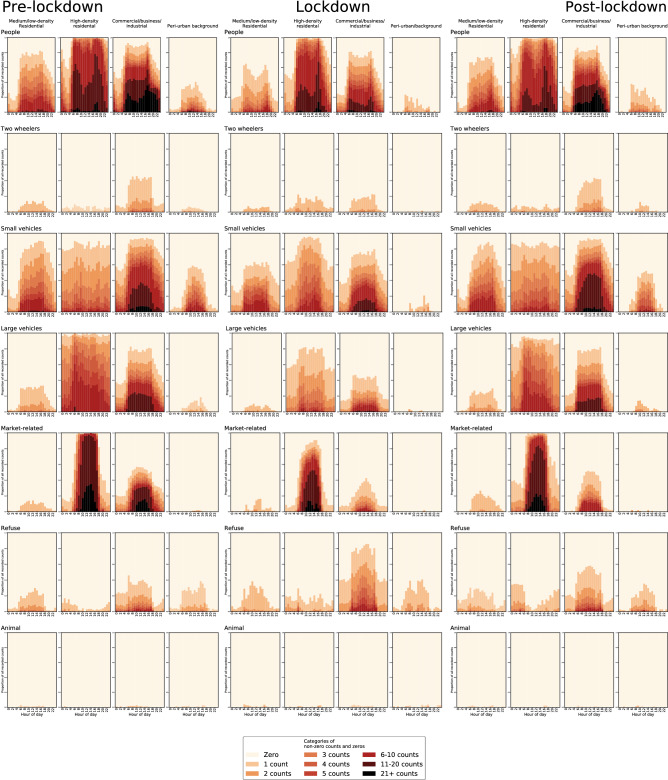
Comparison of object counts before, during and after lockdown. The data are from the eight of the ten fixed sites, which had some data during the city-wide lockdown from the 30th of March to the 20th of April 2020 in response to the COVID-19 pandemic. Pre-lock down refers to the period from the 10th April 2019 to the 29th of March 2020, and post-lockdown from the 21st of April to the 11th June 2020.

During the city-wide lockdown in April 2020, there was a noticeable drop in the number of people, vehicles and market-related objects at CBI sites (Fig. 7). Other research has indicated that people who had non-essential business or could work from home avoided these areas^60^. In contrast, in the high-density settlements and slums, there was little change in the number of people and small vehicles, and more two-wheelers were detected. The number of large vehicles, including tro tros, declined throughout the city, because fewer people commuted for work and business. There were also fewer market-related objects at all sites. This finding is consistent with government advice to shop locally where possible, and relatively strict enforcement of social distancing and hygiene measures for market traders, which other studies indicated led to some traders being removed from the market or entire marketplaces being closed^60^. The number of animals and debris or trash increased slightly in the CBI areas during lockdown. In the two-month period immediately after the lockdown ended, the number and temporal patterns of people and all object categories returned to their pre-lockdown levels, with the exception of market-related objects in CBI areas, which only partially returned from lockdown to pre-lockdown levels (Fig. 7). This trend may be because some people working in the government or the private sector did not (fully) return to their workplace, and may indicate a longer lasting impact from COVID-19 restrictions on the informal market and commercial sector in Accra.

## Discussion

Our unique dataset of spatially and temporally representative images and our contextually-adapted application of computer vision methodology has revealed the patterns and dynamics of the human–environment interface in Accra, a major African city. These novel insights can inform planning and policy decisions for making Accra, and other cities in Africa, more liveable, sustainable, equitable and healthy as they expand and develop. Below, we discuss key contemporary applications of our data and approach in Accra and other cities, and their integration with other sources, that are relevant for health and sustainability.

### Mobility and transport

Our results show that the privately owned and informally operated tro tros were present in large numbers throughout the city’s major roads, while cars and taxis were present on all roads. These patterns arise at least partly because Accra does not have a formal urban public transportation option such as train, tram or even an extensive bus network. The strong correlation between the number of tro tros and the number of people is an indication of their important role in how people move around the city to access jobs and services^55,56^. It also shows their potential impact on population exposure to air and noise pollution, because they tend to be older diesel vehicles, often imported into the country after they were used in wealthier countries^32,33,55,61^, a situation exacerbated by limited enforcement of local emission standards. Similarly, the widespread presence of private cars, whose numbers have increased over time as a growing middle-class has purchased more cars^62^, worsens air and noise pollution and traffic congestion, and may make transportation less equitable among socioeconomic groups^55,63^.

Our data on people and vehicle categories at different times and locations provide a baseline to inform the design of mobility policies, and our image-based system can be used to monitor how they influence traffic volume and traffic fleet composition over time. They may also be used, together with other mobile-phone based mobility data, to indicate where and when public space is most frequently traversed by pedestrians. As we discuss below, these data are also essential inputs for modelling how policies influence traffic patterns, air and noise pollution, and injury risk.

### Environmental pollution, sanitation and waste management

Our data on the spatial and temporal patterns of objects in Accra also help map air and noise pollution and their sources, and guide pollution control policies. For example, in comparing our image-based object count data with previously published measurements of noise levels^64^ and fine particulate matter air pollution (PM_2.5_) concentrations^65^ in Accra, we found that counts of people (Spearman correlation coefficient = 0.48), small vehicles (0.39), large vehicles (0.48), and market-related objects (0.40) were correlated with noise levels (measured in A-weighted decibels (dBA)) recorded within 30 s of the corresponding images collected at the same locations. Weaker correlations were found between co-located PM_2.5_ concentration and people (0.18), small vehicles (0.12), large vehicles (0.13) and market-related objects (0.13), possibly because PM_2.5_ has a combination of local and regional sources. Based on these observations and previous studies^66–68^, we expect that our images and the objects within them can be used as variables within predictive models of time-resolved air and noise pollution. In previous work^69–72^, image data were used to estimate annual average pollution levels, or temporally varying pollution in a limited number of locations. Our temporally representative data serves as a unique basis for such models with spatiotemporal data on human presence, to estimate population exposure to pollution, and how it may be impacted through planning and policy decisions.

Our data also showed that refuse, consisting of trash (discarded items) and debris (remnants of construction materials), was present throughout Accra, albeit at different levels in inner and outer areas, possibly because household refuse is not systematically and regularly collected in the city^73^. The presence and accumulation of refuse increases the risk of flooding, because it blocks open drainage channels which overflow with wastewater or rainwater^9^. These phenomena, as well as the presence of animals which we identified in the city’s peripheries, are also risk factors for vector-borne diseases^11^. Data on refuse and animals could reveal where these risks are most common and identify targets for their control.

### Livelihood and environment in informal settlements

Our results also revealed the extent of human activity in, and the environment of, high-density residential neighbourhoods, many of which are typically classified as informal settlements and slums. In particular, compared to other parts of the city, these neighbourhoods had more human presence and higher volumes of market related activities that are important components of social and commercial networks in African cities and support the livelihood of numerous families^74^. As Accra develops and land use patterns change, it is essential that the social and economic benefits of these activities are protected. Achieving this aim requires careful upgrading of the environment and services to make these areas healthier and more liveable^52,75,76^, while putting in place land tenure arrangements that protect their residents and businesses against displacement.

### Monitoring and evaluation of policies

Our spatiotemporally resolved images dataset and approach provide a model for a digital urban information system that can be used to evaluate the impacts of policy or technical interventions that affect the city’s infrastructure and environment. An example of this potential can be seen by the fact that our approach revealed the substantial changes in the number of people, vehicles and marketplace activity in commercial, business, and industrial areas that coincided with the introduction of pandemic-related lockdown measures, and the return of people and vehicles, but not market activity, to pre-lockdown levels at those same sites. An emerging policy-related application of this approach is to measure the impact of public transport infrastructure, such as a Bus Rapid Transport (BRT) system^77–79^, on traffic volumes and fleet composition in spatially and temporally resolved ways, as has been done in some countries using images from CCTV networks^80,81^, which have recently been also installed in Accra and other African cities^82,83^.

### Strengths and limitations

Our work is among the first applications of computer vision methods for object detection in a city in a low- and middle-income country and especially in Africa. We collected a large number of images at sites representative of, and throughout, the entire city, with fine temporal granularity, which allowed us to assess the dynamics of urban environment and activity over space and time. We adapted deep learning models and training datasets to the specific social and environmental context, for example through selection and labelling of tro tros and market related objects, to enhance the local relevance of the data. We used transfer learning with data augmentation in order to maximise the use of our labelling resources. Our network’s performance was systematically analysed with widely used metrics, providing a benchmark for future global applications of computer vision object detection methods to street view imagery. We covered different land use categories, and stratified our results by these classes, which helps envision how urban development and change may influence cities’ environment and human interactions with the environment. Our approach can be scaled up to other cities, particularly those in West Africa, which have shared features with Accra related to local geography, travel characteristics, social structure and economic activities. The general approach may be replicated using any camera technology capable of capturing time-lapsed images (or video), including CCTV networks that are increasingly deployed in African cities^83^, though appropriate safeguards for privacy are needed for such data. We used open source software, and the weights of our network will be made publicly available, to allow application to other cities, though fine-tuning of the model may be needed to maintain or improve model performance.

Our work also has some limitations. Like any field measurement campaign, there were trade-offs between spatial and temporal granularity of data. Although we had images from over a hundred representative sites across Accra, and the use of stationary cameras allowed us to have temporally resolved images, we did not cover the entire city as data such as Google Street View often do in high-income countries. The number of objects detected within an image is sensitive to the field of view which the cameras capture. Although we implemented systematic positioning and placement of camera height as described in the Methods and Data section, the extent to which objects are visible within the cameras’ field of view may vary between sites and may affect comparison across sites. Finally, although our trained algorithm achieved the target performance threshold, performance varied across objects, with poorer performance for those that were sparsely represented in our training set, like cookstoves and street vendors, or which visually differed from the MS-COCO object categories which the model was pre-trained on, like loudspeakers, and/or have varied visual appearances within the same category, like food. This issue is common in object detection when datasets have uneven frequency across object categories or do not have a unique appearance^84^. Finally, although the Faster R-CNN algorithm, which we used in our transfer learning approach, was amongst the top performing object detection algorithms at time of our analysis, better performing models become available over time, and may improve performance on some object categories^85,86^.

### Conclusions

To select, target and evaluate policies that aim to improve, and reduce inequalities in, health, safety and wellbeing in growing cities in Africa, there is a critical need for spatially and temporally resolved, representative data on human activities and the environment where they take place. Our work shows that systematic collection of image data and application of computer vision techniques can be used in an interdisciplinary approach to complement traditional administrative data sources to reduce the current data gap in cities in Africa and other developing regions and identify key aspects of urban life in a rapidly growing metropolis. Routine collection of images has the potential to complement traditional data platforms and provide governments, researchers, and civil society groups with additional information to identify areas in need of intervention and track the impacts of policies that address them.

## Methods and data

### Field study design for image collection

Over a 15-month period (10th April 2019 to 11th June 2020), we placed cameras at 145 sites throughout GAMA^57^. Sites were set up with permission from residents and owners’ which also helped ensure that the equipment were safe and not interfered with. We operated ten sites for the entire measurement period (referred to as fixed sites throughout the paper) and operated 135 sites for one week each^57^ (referred to as rotating sites). The rotating sites were selected through stratified random sampling from a dataset representing four land cover and neighbourhood classes for the city (20 m $\times$ 20 m resolution)^87^: formal, mostly low- and medium-density residential areas which mostly contained planned road networks; informal, mostly high-density, settlements and slums with small irregular buildings and narrow unpaved roads; commercial, business and industrial (CBI) areas with large buildings used for commercial, industrial, office or warehouse purposes; and other areas that were largely peri-urban or rural, and have relatively dense vegetation (i.e., forest, farmland and grassland), barren land (i.e., sand, soil and dirt) or water.

We oversampled sites in the AMA, which is the main metropolitan centre of GAMA, and where nearly half of the population of GAMA lives. After target locations were selected, we verified their suitability through inspection of aerial images and site visits. The locations of the fixed sites were chosen to cover different land-use classes, areas with different road types and other microenvironmental features, and neighbourhoods with different socioeconomic status and population density. The locations of the fixed sites were as follows: N1 West at Lapaz and Tema Motorway are at the west and east ends of the multi-lane N1 motorway; Asylum Down is on the Central Ring Road; Jamestown and Nima are poor, densely populated neighbourhoods in south and middle of the AMA, respectively; Taifa is an emerging medium-density neighbourhood north of the city; Labadi is an indigenous Ga community along on the Coast; East Legon is an affluent neighbourhood which has a mix of residential space and buildings that house corporate, commercial and small business ventures; Ashaiman is an emerging low-density residential neighbourhood next to the port city of Tema; and University of Ghana Hill is located on top of the quiet, forested Legon Hill and is a part of the university campus. More details on site selection and their assignment to land cover and neighbourhood classes are provided in the study protocol paper^57^.

From the 30th of March 2020 to the 20th of April 2020, Accra imposed a city-wide lockdown due to the COVID-19 pandemic. We have images from eight of the 10 fixed sites for as long as the cameras operated and had memory (median 15 days; 25th–75th percentiles 9–18 days). These data were excluded from our main results on temporal dynamics of object counts. However, in order to examine whether image-based analysis can detect changes in activity patterns, we compared object counts and their hourly variation before, during and after lockdown for these sites.

The study protocol was approved by the University of Ghana Ethics Committee and deemed exempt from full ethics review at Imperial College London and the University of Massachusetts Amherst.

### Image collection hardware

We used the Moultrie-M50 camera trap for taking images. We selected these cameras for several reasons: First, they had sufficient memory for logging data, battery life, and were rugged so as to withstand weather conditions such as heavy rainstorms, humidity, heat and seasonal dust storms, while capturing high-quality data. Second, the cameras are programmable to take images at fixed intervals and, importantly, automatically switch to night-vision mode when it is dark which allows having data at night as well as day. Third, the cameras capture images with 20-megapixel quality in a 36.7° field of view, which is sufficient resolution for identifying features from the street-level with object detection, whilst allowing storage within camera memory for duration of measurement, and regular back-ups and upload to a data server. We programmed each camera to take a time-stamped image every five minutes which allowed the camera memory to store an entire week of images (n ~ 2000). Images were also captured at night in black and white with infrared flash. These qualities helped address logistical constraints of conducting robust high-quality environmental monitoring in a city with unreliable electricity supply and extreme weather. Additional details on camera specifications are available in the study protocol^57^.

We deployed cameras in weather protective cases, and affixed them to trees or poles which were either directly in the ground or on a rooftop or balcony (Fig. 1). The target installation height was at ~ 4 m above the ground. However, in practice we had to be within ± 1 m of the target height for logistical reasons. The cameras were mounted in metal protective cases with rotational multi-access brackets for ease of orientation on the outside of a box holding air and noise pollution measurement equipment (Fig. 1). We identified appropriate angles of view for each camera by assessing and adjusting its image angle using a tablet computer. The cameras were oriented such that they captured the public street view to incorporate the main thoroughfare. Some measurement sites had two cameras (90 sites) while others had one (55 sites), based on whether one or two fields of view would be needed to capture the public streetscape. Cameras were placed at all fixed sites and 4–5 rotating sites each week. For the rotating sites, we returned 7-days after initial set-up to take down the equipment and bring it to the lab for cleaning and data download. Fresh equipment was then re-deployed 48 h later at new sites. For the fixed sites, we brought replacement batteries and Secure Digital (SD) cards to the site so as not to have a disruption in continuous monitoring. Lastly, we asked the owner or resident of each site to call a member of the team if they noticed any problems with the equipment.

While we aimed to capture images at all target measurement sites and for the entire measurement period, in some cases, camera equipment failed or shut off due to stress from high temperature or a similar factor, or breakage from prolonged wear and tear. There was also a two-week interruption in continuous fixed-site monitoring in January 2020 due to a scheduled equipment quality control check. Detailed information on the missingness of images is provided in Supplementary Fig. 1.

### Selection and labelling of objects in images

We used a systematic interdisciplinary consensus process to identify the objects relevant for various domains in urban environmental research. We first constructed a sample set of 100 images consisting of 10 images from each fixed site. Twenty-four researchers, from social and environmental sciences, urban planning, public health and machine learning, and from countries in Africa, Asia, Europe and North America independently reviewed the images and listed visual features relevant to mobility, safety, leisure and play, daily life activities like shopping, air and noise pollution and sanitation and hygiene. The reviewers were not explicitly informed on what is detectable using computer vision methods. This process led to identification of 113 unique features listed in the Supplementary Information S1.

Since our images were captured in space and time, we filtered these features to only include non-stationary objects. These are singly identifiable and countable “things”, with clear visual boundaries that could change in location or frequency of presence across time, e.g., cars but not trees (which are stationary) or grass (which is both stationary and not countable). After this process, 69 candidate objects remained. Nine of the study researchers independently scored these 69 objects on a scale from one to five on the following attributes: uniqueness compared to other listed objects, frequency as perceived from the sample images and utility or relevance from the perspective of urban environment, health and wellbeing research and policy. The mean for each attribute was calculated across all nine respondents, and the final score for each object was the average of its three attribute means. Empirically, mean frequency score was positively correlated with mean utility score across these 69 objects (Pearson correlation coefficient = 0.84).

We selected the top 20 scoring objects for labelling. We made one post-hoc alteration to the list: combining livestock, which was in the top 20, with goat and dog, which were in the subsequent 15, to create a combined category of animal. The 20 selected objects were: person, market vendor (a person carrying a container over their heads which is a common scene in Accra and other African markets), car, taxi, pick-up truck, bus, lorry, van, tro tro, motorcycle, bicycle, market stall, loudspeaker, umbrella (commonly used to protect market and roadside vendors from the sun and rain), cookstove, cooking pot/bowl (which frequently contain wares for sale in the marketplace), food, trash, (piece of) debris and animal. Examples of these objects can be seen in Fig. 2. For presentation, these objects are grouped into the following categories: people (persons and market vendors); large vehicles (lorries, vans, buses and tro tros); small vehicles (cars, taxis and pick-up trucks); two wheelers (bicycles and motorcycles); objects from the market and street vending which are common in African cities^5,6^ (market stalls, umbrellas, cookstoves, cooking pots/bowls, food and loudspeakers); refuse (trash and debris); and animals. Members of the research team identified and labelled these objects in bounding boxes across 1,250 images in a process described in SI S2.

### Retraining of deep convolutional neural network with transfer learning

Training a CNN for object detection from scratch requires hundreds of thousands of labelled images^88^ which is not feasible for most applications. Therefore, most object detection applications retrain a pre-trained network using a smaller amount of data, a process known as transfer learning^89–91^. For this work, we retrained the Faster R-CNN model^92^ (atrous convolutions with an Inception V2 base), which is provided by the Tensorflow Object Detection API V1^93^, with emphasis on enhancing performance for detecting objects that are seen in an African city like Accra. We used this model for two reasons. First, it had one of the best performances of any network when tested on a benchmark dataset at the time of model selection^93^, while not having excessively high memory requirement, hence balancing accuracy and efficiency. Second, the model was pre-trained on the Microsoft Common Objects in Context (MS-COCO) dataset^94^, which contains 91 object categories, some of which overlapped with those in our study, e.g., person, umbrella, bus, truck, car, bicycle, motorcycle, and multiple categories of animals and foods. By comparison, in our work, all food and animal types were grouped, while different vehicle types relevant to Accra’s context (e.g., tro tro and taxi) were distinguished. Other locally relevant objects such as trash, debris, market stalls, street vendors, cookstoves and loudspeakers, which were on our list, are not available in common computer vision datasets. We also tried a CNN with the same architecture as Faster R-CNN pre-trained on the Google OpenImages (V4) dataset^95^ but the performance of the retrained network on our images, as measured by mean Average Precision (mAP), was worse than that pre-trained on MS-COCO.

We retrained the Faster R-CNN model for our object detection task in the following manner. First, we simultaneously stratified the images by frequency and size (as measured by pixel count) of each object category in the image, and colour versus greyscale (which correspond with day and night time images), and split the strata into 60–20-20% (750–250-250 image) subsets for training, validation and testing as described in SI S3. In this way, objects in each category were represented as evenly as possible in all three sets, and under similar visibility conditions. The resulting validation set was used as described below to set training features (e.g., image augmentation operations) and hyperparameters (e.g., learning rate) that would optimise performance. Finally, the test set was used to evaluate the performance of the model (trained on the combined 1000 images of training and validation data) once all features of training had been configured. The retrained CNN was then applied to the entire image set to identify and locate the candidate objects in each image (Fig. 2).

### Data augmentation and optimisation of training approach and hyperparameters

We used two types of data augmentation, described in SI S4, an approach that helps the trained network to avoid overfitting and identify objects in a broader set of conditions^96,97^. We optimised the training approach and hyperparameters as described in SI S5. The optimisation process yielded a 20% improvement in mAP compared with no augmentation or changes to the default learning schedule and maximum proposal number provided in Tensorflow Object Detection API V1. Object-specific improvements in mAP are shown in Supplementary Table 1.

### Performance of the retrained network

After finalizing our training approach and parameters, we trained the algorithm on the combined 1000 images of the training and validation sets and evaluated on the 250-image test set which had been entirely held back from the model as described in SI S6.

### Fixed site data down-sampling

We had approximately five times more images from fixed sites than from rotating sites. When reporting site-specific results (Figs. 4 and 5), we report object counts per image which eliminates the impact of different image numbers. For presenting hourly variation (Figs. 6, 7 and Supplementary Fig. 3), which combine data across sites, we down-sampled object count data from the fixed site camera images, such that each fixed site camera's contribution to the figures comprises an equivalent amount of data as a camera from a rotating site. For each object, we down-sampled by ordering the images within every hourly interval from each fixed site by the counts of objects, from lowest to highest, and selecting a subset of images from equally spaced quantiles of object counts such that the total number for each fixed site was approximately equal to the 2,016 images collected at the rotating sites. Ordered, stratified down-sampling better preserves the distribution of object counts in the fixed site images than simple random sampling. This process is described in detail in SI 7.

### Ethical approval

The study protocol was approved by the University of Ghana Ethics Committee and deemed exempt from full ethics review at Imperial College London and the University of Massachusetts Amherst.

## Supplementary Information

Supplementary Information.

## Data Availability

Our image labelling protocol, analysis code, trained object detection model, object count data and site metadata can be downloaded from http://globalenvhealth.org/code-data-download/ and http://equitablehealthycities.org/data-download/ upon publication of the paper. Requests for re-analysis of images should be sent to the corresponding authors.

## References

[1] Ezzati M (2018). Cities for global health. BMJ.

[2] Glazener A (2021). Fourteen pathways between urban transportation and health: A conceptual model and literature review. J. Transp. Health.

[3] Sowatey E (2018). Spaces of resilience, ingenuity, and entrepreneurship in informal work in Ghana. Int. Plan. Stud..

[4] Beek J, Thiel A (2017). Orders of trade: regulating Accra’s Makola market. J. Leg. Plur. Unoff. Law.

[5] Solomon-Ayeh, B. E., King, R. S. & Decardi-Nelson, I. *Street Vending and the Use of Urban Public Space in Kumasi, Ghana*. (2011).

[6] Brown A, Lyons M, Dankoco I (2010). Street traders and the emerging spaces for urban voice and citizenship in African cities. Urban Stud..

[7] Karley N (2009). Flooding and physical planning in urban areas in West Africa: Situational analysis of Accra, Ghana. Theor. Empir. Res. Urban Manag..

[8] Honingh D (2020). Urban river water level increase through plastic waste accumulation at a rack structure. Front. Earth Sci..

[9] Douglas I (2008). Unjust waters: Climate change, flooding and the urban poor in Africa. Environ. Urban..

[10] Moulds, S., Buytaert, W., Templeton, M. R. & Kanu, I. Modeling the impacts of urban flood risk management on social inequality. *Water Resour. Res.* **57**, e2020WR029024 (2021). 10.1029/2020WR029024 PMC7615406 38130829

[11] Grimes JE (2015). The roles of water, sanitation and hygiene in reducing schistosomiasis: a review. Parasit. Vectors.

[12] Johnson SAM (2016). Myiasis in dogs in the Greater Accra Region of Ghana. Vector-Borne Zoonotic Dis..

[13] United Nations, Department of Economic and Social Affairs, & Population Division. *World urbanization prospects: the 2018 revision*. (2019).

[14] ARUP and Cities Alliance. Future Proofing Cities Metropolitan Cities in Ghana. (2016).

[15] Daramola A, Ibem EO (2010). Urban environmental problems in Nigeria: implications for sustainable development. J. Sustain. Dev. Afr..

[16] Lall, S. V., Henderson, J. V. & Venables, A. J. *Africa’s Cities : Opening Doors to the World*. (World Bank, 2017).

[17] Randall S (2015). UN Census “Households” and Local Interpretations in Africa Since Independence. SAGE Open.

[18] Randall S, Coast E (2015). Poverty in African households: The Limits of Survey and Census Representations. J. Dev. Stud..

[19] Soomro K, Bhutta MNM, Khan Z, Tahir MA (2019). Smart city big data analytics: An advanced review. WIREs Data Min. Knowl. Discov..

[20] Joubert A, Murawski M, Bick M (2021). Measuring the big data readiness of developing countries—Index development and its application to Africa. Inf. Syst. Front..

[21] Kwan M-P (2016). Algorithmic geographies: Big data, algorithmic uncertainty, and the production of geographic knowledge. Ann. Am. Assoc. Geogr..

[22] Yang D, Qu B, Cudre-Mauroux P (2021). Location-centric social media analytics: Challenges and opportunities for smart cities. IEEE Intell. Syst..

[23] Yang, J., Hauff, C., Houben, G.-J. & Bolivar, C. T. Diversity in Urban Social Media Analytics. in *Web Engineering* (eds. Bozzon, A., Cudre-Maroux, P. & Pautasso, C.) 335–353 (Springer International Publishing, 2016). 10.1007/978-3-319-38791-8_19.

[24] GSM Association. The Mobile Economy Sub-Saharan Africa. (2021).

[25] Batran M, Mejia MG, Kanasugi H, Sekimoto Y, Shibasaki R (2018). Inferencing human spatiotemporal mobility in Greater Maputo via mobile phone big data mining. ISPRS Int. J. Geo-Inf..

[26] Kung KS, Greco K, Sobolevsky S, Ratti C (2014). Exploring universal patterns in human home-work commuting from mobile phone data. PLoS ONE.

[27] Wesolowski A, O’Meara WP, Eagle N, Tatem AJ, Buckee CO (2015). Evaluating spatial interaction models for regional mobility in sub-Saharan Africa. PLOS Comput. Biol..

[28] Jay J (2020). Neighbourhood income and physical distancing during the COVID-19 pandemic in the United States. Nat. Hum. Behav..

[29] Shi W, Zhang A, Zhou X, Zhang M (2018). Challenges and prospects of uncertainties in spatial big data analytics. Ann. Am. Assoc. Geogr..

[30] Blumenstock J, Cadamuro G, On R (2015). Predicting poverty and wealth from mobile phone metadata. Science.

[31] Blumenstock J (2018). Don’t forget people in the use of big data for development. Nature.

[32] Arku RE (2015). Personal particulate matter exposures and locations of students in four neighborhoods in Accra, Ghana. J. Expo. Sci. Environ. Epidemiol..

[33] Dionisio KL (2010). Within-neighborhood patterns and sources of particle pollution: Mobile monitoring and geographic information system analysis in four communities in Accra. Ghana. Environ. Health Perspect..

[34] Samadi Z, Yunus RM, Omar D, Bakri AF (2015). Experiencing urban through on-street activity. Procedia - Soc. Behav. Sci..

[35] Glaeser EL, Kominers SD, Luca M, Naik N (2018). Big data and big cities: The promises and limitations of improved measures of urban life. Econ. Inq..

[36] Goel R (2018). Estimating city-level travel patterns using street imagery: A case study of using Google Street View in Britain. PLoS ONE.

[37] Ibrahim MR, Haworth J, Cheng T (2020). Understanding cities with machine eyes: A review of deep computer vision in urban analytics. Cities.

[38] Weichenthal S, Hatzopoulou M, Brauer M (2019). A picture tells a thousand…exposures: Opportunities and challenges of deep learning image analyses in exposure science and environmental epidemiology. Environ. Int..

[39] Biljecki F, Ito K (2021). Street view imagery in urban analytics and GIS: A review. Landsc. Urban Plan..

[40] Rzotkiewicz A, Pearson AL, Dougherty BV, Shortridge A, Wilson N (2018). Systematic review of the use of Google Street View in health research: Major themes, strengths, weaknesses and possibilities for future research. Health Place.

[41] Suel E, Polak JW, Bennett JE, Ezzati M (2019). Measuring social, environmental and health inequalities using deep learning and street imagery. Sci. Rep..

[42] Time to discover new places in Africa. Ghana, Senegal and Uganda now on Street View! *Official Google Africa Blog.* https://africa.googleblog.com/2017/02/time-to-discover-new-places-in-africa.html.

[43] Krylov VA, Kenny E, Dahyot R (2018). Automatic discovery and geotagging of objects from street view imagery. Remote Sens..

[44] Zhao Z-Q, Zheng P, Xu S-T, Wu X (2019). Object Detection With Deep Learning: A Review. IEEE Trans. Neural Netw. Learn. Syst..

[45] Yin L, Cheng Q, Wang Z, Shao Z (2015). ‘Big data’ for pedestrian volume: Exploring the use of Google Street View images for pedestrian counts. Appl. Geogr..

[46] Liu, J., Zhang, S., Wang, S. & Metaxas, D. Multispectral Deep Neural Networks for Pedestrian Detection. in *Procedings of the British Machine Vision Conference 2016* 73.1–73.13 (British Machine Vision Association, 2016). doi:10.5244/C.30.73.

[47] Rahman, M. M., Sainju, A. M., Yan, D. & Jiang, Z. Mapping Road Safety Barriers Across Street View Image Sequences: A Hybrid Object Detection and Recurrent Model. in *Proceedings of the 4th ACM SIGSPATIAL International Workshop on AI for Geographic Knowledge Discovery* 47–50 (Association for Computing Machinery, 2021).

[48] Fan, Q., Brown, L. & Smith, J. A closer look at Faster R-CNN for vehicle detection. in *2016 IEEE Intelligent Vehicles Symposium (IV)* 124–129 (2016). 10.1109/IVS.2016.7535375.

[49] Campbell, A., Both, A. & Sun, Q. (Chayn). Detecting and mapping traffic signs from Google Street View images using deep learning and GIS. *Comput. Environ. Urban Syst.* **77**, 101350 (2019).

[50] DeVries, T., Misra, I. & Wang, C. Does Object Recognition Work for Everyone? *Proc. IEEECVF Conf. Comput. Vis. Pattern Recognit. CVPR Workshop* 52–59.

[51] Ghana Statistical Service. Greater Accra Population. (2020).

[52] World Bank. Rising through Cities in Ghana : Ghana Urbanization Review Overview Report. (2015).

[53] Clark SN (2021). Small area variations and factors associated with blood pressure and body-mass index in adult women in Accra, Ghana: Bayesian spatial analysis of a representative population survey and census data. PLOS Med..

[54] Bixby H (2022). Quantifying within-city inequalities in child mortality across neighbourhoods in Accra, Ghana: a Bayesian spatial analysis. BMJ Open.

[55] Musah BI, Peng L, Xu Y (2020). Urban Congestion and Pollution: A Quest for Cogent Solutions for Accra City. IOP Conf. Ser. Earth Environ. Sci..

[56] Birago, D., Opoku Mensah, S. & Sharma, S. Level of service delivery of public transport and mode choice in Accra, Ghana. *Transp. Res. Part F Traffic Psychol. Behav.* **46**, 284–300 (2017).

[57] Clark, S. N. *et al.* High-resolution spatiotemporal measurement of air and environmental noise pollution in Sub-Saharan African cities: Pathways to Equitable Health Cities Study protocol for Accra, Ghana. *BMJ Open* **10**, 1 (2020). 10.1136/bmjopen-2019-035798 PMC7440835 32819940

[58] Gough KV (2010). Continuity and adaptability of home-based enterprises: A longitudinal study from Accra, Ghana. Int. Dev. Plan. Rev..

[59] Rooney MS (2012). Spatial and temporal patterns of particulate matter sources and pollution in four communities in Accra, Ghana. Sci. Total Environ..

[60] Asante LA, Mills RO (2020). Exploring the Socio-Economic Impact of COVID-19 Pandemic in Marketplaces in Urban Ghana. Afr. Spectr..

[61] Zhou Z (2013). Chemical composition and sources of particle pollution in affluent and poor neighborhoods of Accra, Ghana. Environ. Res. Lett..

[62] Senadza B, Never B, Kuhn S, Asante FA (2020). Profile and determinants of the middle classes in Ghana: Energy use and sustainable consumption. J. Sustain. Dev..

[63] Urban Age Programme. Cities and Social Equity - Reports. https://urbanage.lsecities.net/reports/cities-and-social-equity#3-three-perspectives-on-inequality (2009).

[64] Clark SN (2021). Space-time characterization of community noise and sound sources in Accra, Ghana. Sci. Rep..

[65] Alli, A. S. *et al.* Spatial-temporal patterns of ambient fine particulate matter (PM2.5) and black carbon (BC) pollution in Accra. *Environ. Res. Lett.* **16**, 074013 (2021). 10.1088/1748-9326/ac074a PMC8227509 34239599

[66] Forehead H, Huynh N (2018). Review of modelling air pollution from traffic at street-level - The state of the science. Environ. Pollut..

[67] Sharma A, Bodhe GL, Schimak G (2014). Development of a traffic noise prediction model for an urban environment. Noise Health.

[68] Tang UW, Wang ZS (2007). Influences of urban forms on traffic-induced noise and air pollution: Results from a modelling system. Environ. Model. Softw..

[69] Ganji A, Minet L, Weichenthal S, Hatzopoulou M (2020). Predicting traffic-related air pollution using feature extraction from built environment images. Environ. Sci. Technol..

[70] Hong KY, Pinheiro PO, Weichenthal S (2020). Predicting outdoor ultrafine particle number concentrations, particle size, and noise using street-level images and audio data. Environ. Int..

[71] Qi M, Hankey S (2021). Using street view imagery to predict street-level particulate air pollution. Environ. Sci. Technol..

[72] Suel E (2022). What you see is what you breathe? Estimating air pollution spatial variation using street-level imagery. Rem. Sens..

[73] Yoada RM, Chirawurah D, Adongo PB (2014). Domestic waste disposal practice and perceptions of private sector waste management in urban Accra. BMC Public Health.

[74] Owusu, G., Agyei-Mensah, S. & Lund, R. Slums of hope and slums of despair: Mobility and livelihoods in Nima, Accra. *Nor. Geogr. Tidsskr. - Nor. J. Geogr.* **62**, 180–190 (2008).

[75] Ezeh A (2017). The history, geography, and sociology of slums and the health problems of people who live in slums. The Lancet.

[76] Turley R, Saith R, Bhan N, Rehfuess E, Carter B (2013). Slum upgrading strategies involving physical environment and infrastructure interventions and their effects on health and socio-economic outcomes. Coch. Database Syst. Rev..

[77] Agyemang, E. The bus rapid transit system in the Greater Accra Metropolitan Area, Ghana: Looking back to look forward. *Nor. Geogr. Tidsskr. - Nor. J. Geogr.* **69**, 28–37 (2015).

[78] Citi FM. Aayalolo buses to ply Adenta-Accra route—Minister. *Citi 97.3 FM - Relevant Radio. Always* https://citifmonline.com/2017/03/aayalolo-buses-to-ply-adenta-accra-route-minister/ (2017).

[79] Ministry of Transport Greater Accra Regional Coordinating Council. Transportation Master Plan: Greater Accra Region (Final Report). (2016).

[80] Peppa MV (2021). Towards an end-to-end framework of CCTV-based urban traffic volume detection and prediction. Sensors.

[81] Fedorov A, Nikolskaia K, Ivanov S, Shepelev V, Minbaleev A (2019). Traffic flow estimation with data from a video surveillance camera. J. Big Data.

[82] Palinwinde Jacobs, D. Activate CCTV cameras installed in Accra to curb crime—Okoe Vanderpuije. *Citinewsroom - Comprehensive News in Ghana* (2021).

[83] Jili B (2022). Africa: Regulate surveillance technologies and personal data. Nature.

[84] Ouyang, W., Wang, X., Zhang, C. & Yang, X. Factors in Finetuning Deep Model for Object Detection With Long-Tail Distribution. in 864–873 (2016).

[85] Bochkovskiy, A., Wang, C.-Y. & Liao, H.-Y. M. *YOLOv4: Optimal Speed and Accuracy of Object Detection*. https://github.com/AlexeyAB/darknet. (2020).

[86] Tan, M., Pang, R. & Le, Q. V. EfficientDet: Scalable and Efficient Object Detection. in *2020 IEEE/CVF Conference on Computer Vision and Pattern Recognition (CVPR)* 10778–10787 (2020). doi:10.1109/CVPR42600.2020.01079.

[87] World Bank Group. 2014 Land Cover Classification of Accra, Ghana. https://datacatalog.worldbank.org/search/dataset/0039825/c--2014-Land-Cover-Classification-of-Accra--Ghana (2014).

[88] LeCun Y, Bengio Y, Hinton G (2015). Deep learning. Nature.

[89] Pan SJ, Yang Q (2010). A Survey on Transfer Learning. IEEE Trans. Knowl. Data Eng..

[90] Tan, C. *et al.* A Survey on Deep Transfer Learning. in *Artificial Neural Networks and Machine Learning—ICANN 2018* (eds. Kůrková, V., Manolopoulos, Y., Hammer, B., Iliadis, L. & Maglogiannis, I.) 270–279 (Springer International Publishing, 2018). 10.1007/978-3-030-01424-7_27.

[91] Yosinski, J., Clune, J., Bengio, Y. & Lipson, H. How transferable are features in deep neural networks? in *Proceedings of the 27th International Conference on Neural Information Processing Systems - Volume 2* 3320–3328 (MIT Press, 2014).

[92] Ren S, He K, Girshick R, Sun J (2017). Faster R-CNN: Towards real-time object detection with region proposal networks. IEEE Trans. Pattern Anal. Mach. Intell..

[93] Huang, J. *et al.* Speed/Accuracy Trade-Offs for Modern Convolutional Object Detectors. in *2017 IEEE Conference on Computer Vision and Pattern Recognition (CVPR)* 3296–3297 (IEEE, 2017). 10.1109/CVPR.2017.351.

[94] Lin, T.-Y. *et al.* Microsoft COCO: Common Objects in Context. in *Computer Vision—ECCV 2014* (eds. Fleet, D., Pajdla, T., Schiele, B. & Tuytelaars, T.) 740–755 (Springer International Publishing, 2014). 10.1007/978-3-319-10602-1_48.

[95] Kuznetsova A (2020). The open images dataset V4. Int. J. Comput. Vis..

[96] Shorten C, Khoshgoftaar TM (2019). A survey on Image Data Augmentation for Deep Learning. J. Big Data.

[97] Zoph, B. *et al.* Learning Data Augmentation Strategies for Object Detection. in *Computer Vision—ECCV 2020* (eds. Vedaldi, A., Bischof, H., Brox, T. & Frahm, J.-M.) vol. 12372 566–583 (Springer International Publishing, 2020).

[98] U.S. Geological Survey. Landsat-8 imagery. (2020).

