# Supplementary material for: Characterisation of urban environment and activity across space and time using street images and deep learning in Accra
Source: Sci Rep. 2022 Nov 28;12:20470. doi: 10.1038/s41598-022-24474-1 (PMC9703424; doi:10.1038/s41598-022-24474-1)
Supplement: Supplementary file 1 — Supplementary Information. [file 41598_2022_24474_MOESM1_ESM.pdf]

Characterisation of urban environment and activity across space and time using street images and deep learning in Accra

Supplementary Information

Ricky Nathvani*[1,2], Sierra N Clark*[1,2], Emily Muller[1,2], Abosede S Alli[3], James E Bennett[1,2], James Nimo[4], Josephine Bedford Moses[4], Solomon Baah[4], A Barbara Metzler[1,2], Michael Brauer[5], Esra Suel[1,6], Allison Hughes[4], Theo Rashid[1,2], Emily Gemmel[5], Simon Moulds[7], Jill Baumgartner[8,9], Mireille Toledano[1,2,10], Ernest Agyemang[11], George Owusu[12], Samuel Agyei-Mensah[11], Raphael E Arku[†3], Majid Ezzati[†1,2,13]

* Joint first authors

† Joint senior authors

[1] Department of Epidemiology and Biostatistics, School of Public Health, Imperial College London, London, UK

[2] MRC Centre for Environment and Health, School of Public Health, Imperial College London, London, UK

[3] Department of Environmental Health Sciences, School of Public Health and Health Sciences, University of Massachusetts, Amherst, USA

[4] Department of Physics, University of Ghana, Accra, Ghana

[5] School of Population and Public Health, The University of British Columbia, Vancouver, Canada

[6] Swiss Data Science Centre, ETH Zurich, Zurich, Switzerland

[7] Department of Civil and Environmental Engineering, Imperial College London, London, UK

[8] Institute for Health and Social Policy, McGill University, Montreal, Canada

[9] Department of Epidemiology, Biostatistics, and Occupational Health, McGill University,

Montreal, Canada

[10] Mohn Centre for Children's Health and Wellbeing, School of Public Health, Imperial College London, London, UK

[11] Department of Geography and Resource Development, University of Ghana, Accra, Ghana

[12] Institute of Statistical, Social & Economic Research, University of Ghana, Accra, Ghana

[13] Regional Institute for Population Studies, University of Ghana, Accra, Ghana

S1. Features identified from the initial review of images

As stated in the main paper, the first step of the interdisciplinary consensus process of object identification involved reviewing 100 images and listing visual features relevant to mobility, safety, leisure and play, daily life activities like shopping, air and noise pollution and sanitation and hygiene. The 24 reviewers collectively listed the following 113 features:

Air conditioning unit; air pressure; aircraft; ambulance; animal; asbestos; ATV (all-terrain vehicle/Quadbike); bicycle; billboard; bird; building; bus; bus station (presence of); bush; car; cart; cat; cell tower; chair; chimney; clothes; clouds; construction site (presence of); cooking bowl; day/night time; debris; dog; door; drain; dust; electric wire; factory; factory (presence of); fence; field (presence of); fire; flat surface (presence of); food; goat; grass; haze (presence of); house; industrial engine; “keep off grass” sign; landfill; lawn; livestock; lorry; lorry station (presence of); loudspeaker; manhole; mannequin; market (presence of); motorcycle; musical instruments; open gutter; park (presence of); person; playing field (presence of); post; poster; pothole; potted plant; public transport (presence of); pylon; rain (presence of); road; road barrier; road guard rail; road sign; roof; shade (presence of); sheep; ship; shoes; sidewalk; skateboard; sky; smoke; stall; state of weather; cookstove; street light; table; tap; taxi; temperature; toilet; traffic; traffic light; train; trash; trash can; tree; tricycle; trolley; tro tro; truck; TV antenna; umbrella; unoccupied land (presence of); van; vegetation; vehicle (presence of); vendor; visibility; wall; water; water tank; wind; wind passages; window.

S2. Labelling of objects in images

In a pilot test, two of the authors (RN and EM) labelled two objects, namely person and car, in 200 of our images, to establish feasibility of labelling and determine the time needed for labelling. We decided to label on the order of 10^3 images ($O(10^3)$) for transfer learning – i.e., (re)training and testing of a pre-trained convolutional neural network (CNN) – to balance performance and the time needed for labelling by the research team as described in the next section. Prior work has demonstrated that transfer learning with $O(10^3)$ images allows a retrained CNN to have sufficiently good performance for object detection[1,2]. Increasing the number of images improves performance but at a slower rate[3], which was also the case in our data as described below. We decided on an initial set of 1,250 images for training and evaluating the model, which led to good performance as we describe in detail below. These 1,250 images were drawn as a stratified sample from the 95 sites that had been operated at the time of labelling, evenly split between colour and greyscale images.

Eleven of the authors labelled objects in these 1,250 images with a bounding box using LabelBox, an online labelling platform. Labellers were given visual examples of each object and thorough instruction to enhance consistency of labelling under different conditions (e.g., partially obscured and overlapping objects). Over the two-month labelling period, labellers were given feedback and all labels were reviewed by two of the authors (RN or EM) to ensure consistency with the guidelines before being accepted into the final set. In addition, thirteen images were re-labelled by multiple researchers against a benchmark set to evaluate labelling consistency and quality. The median correlation between labellers and benchmark labels for the number of objects identified was 0.84 (interquartile range 0.79-0.88). The average percentage overlap between the area of the bounding boxes in all benchmark labels and the labellers' labels for all object categories was 44%. The final 1,250 labelled images contained 10,694 identified instances of the above 20 objects.

S3. Stratification of images into training, validation and testing sets

We used a genetic algorithm for stratification of images to maximise even proportions in the training, validation and testing datasets for all object categories, simultaneously stratified by frequency, size, and colour versus greyscale images. Size categorisations were based on those used for MS-COCO, with small, medium and large objects defined as those of $<32^2$, 32^2 to $<96^2$, and $\geq 96^2$ pixels, respectively. In this algorithm, "population members" consist of a sequence of 1,250 non-repeating indexes, with each index each referring to a different image (represented by the integers 1 to 1,250). Initially, the first 1,000 indices were designated to the combined training and validation sets, and the final 250 to the test set. We began with a randomly initialised population of 7,500 members. These members underwent crossovers (random sub-setting and combination of any two members) and mutations (random reshuffling of some indices in a member), followed by fitness evaluation, such that each new population member contains exactly one copy of each labelled image. The fitness of each member was determined by how close the proportion of each object's counts, separated by size class, match the ideal proportion in each set (e.g., whether the fraction of all small bicycles in the 1,000 training and validation image set is close to its 80% share of all images), as well as the proportion of colour and black and white images. This process was then repeated 100 times (or generations) with the best performing population member selected to split the 1,250 images into a 1,000-image training-plus-validation set and 250-image test set. The entire process was then repeated to split the 1,000 training-plus-validation images into the 750-image training set and 250-image validation set with an even distribution of objects. This approach ensured that even objects such as cookstoves and loudspeakers, which numbered in only 18 and 17 instances, respectively, across all 1,250 images, were distributed as evenly as possible across the training, validation and testing splits, under similar visibility conditions. The genetic algorithm was implemented using the Python library

DEAP[4].

S4. Data augmentation

As stated in the main paper, we used two types of data augmentation to avoid over-fitting during training and identify objects in a broader set of conditions. The first augmentation strategy, implemented in Tensorflow Object Detection API V1[5], involved simultaneously applying a range of image level transformations that broadly corresponded to diverse circumstances that commonly occur in image datasets like ours that are captured over space and time. Examples include switching colour images to greyscale, changing brightness and hue, and cropping images which reflect variations in factors like time of day, weather, angle and the image-taking environment (e.g., obstructions). These were applied independently, each with the default probabilities of the Tensorflow Object Detection API V1, which are all <0.5. The second form of augmentation involved a learned augmentation policy[6] that itself combines several image level transformations which have been empirically shown to improve learning for the purpose of object detection in diverse data sets. We implemented this strategy in combination with augmentation with random Gaussian patches[7], which probabilistically applies a pixel-level noise with a Gaussian distribution to images and has been shown to improve out of sample performance of object detection models[7]. Using both augmentation strategies improved performance in our analysis compared to no augmentation or either data augmentation strategy in isolation.

S5. Optimisation of training approach and hyperparameters

We first determined the data augmentation strategy, learning schedule and the number of proposals (initial suggestions or priors for object locations) used by the model by training on the 750 images designated for training and measuring, and iteratively improving, out of sample performance estimated on the 250 images designated as the validation set.

Training was performed on full resolution 1920×1009 pixel JPEG format images using an NVIDIA QUADRO RTX 6000 GPU with Tensorflow Object Detection API V1. The algorithm was first trained with a Momentum optimizer and initial learning rate of 0.0003 for eight epochs without any augmentation, followed by eight epochs with the first augmentation strategy. Then it was trained with the second augmentation strategy for eight epochs with a learning rate of 0.0003, then 23 epochs at a learning rate of 0.00003 and finally at a learning rate of 0.000003 until the mAP, which was our metric of model performance as described below, no longer improved (nine further epochs).

The optimal number of proposals for the first stage of the Faster R-CNN detector, found via grid search, was 500 compared to the default of 300. Furthermore, retraining performance (measured by validation set mAP) was highest when adopting the same learning rate as used during the original training procedure on the MS-COCO dataset (8 epochs of 0.0003, 23 epochs at 0.00003, and then an indefinite number of epochs at 0.000003), compared to smaller learning rates which are used in some transfer learning applications. This may be because our images differ from those of the MS-COCO dataset the algorithm was initially trained on, requiring larger updates of parameters in the initial stages of training to learn features relevant to the new dataset.

S6. Measurement of model performance

We report mean average precision (mAP), which measures whether the network accurately identifies both the presence of an object and localises its location and size, as represented by its boundaries[8]. A true positive is defined when the predicted bounding box overlaps with the ground truth box above a range of intersection-over-union (the overlapping area between two bounding boxes divided by the area of their union) thresholds, from 0.50 to 0.95 in 0.05 intervals, and is identified as the correct object category. Identification is defined as when the final layer object classifier produces a confidence score above a range of thresholds for a given object category. Precision is defined as the proportion of bounding boxes that are true positives while recall is the fraction of true-positive detections as a proportion of all ground truth boxes. The average precision is the area under the precision-recall curve, obtained from varying the confidence score thresholds. The mean refers to the mean taken across different intersection-over-union overlap thresholds, and confidence scores either for individual object categories or across object categories. This metric was originally constructed to evaluate performance of diverse models on the MS-COCO dataset[9].

At the time of model selection, state-of-the-art performance mAP for a model on a subset of MS-COCO data was ~0.35, and median performance among commonly used models on this dataset was ~0.2. Transfer learning is able to improve performance mAP by ~0.1 as compared with training from scratch[3]. Consistent with the commonly used models, we set a target mAP of 0.2 for our fine-tuned network. This choice also takes into account some of the detection challenges posed by our data, compared to a dataset like MS-COCO. Specifically, images such as those in the MS-COCO dataset that were collated for the purpose of training an object detection model generally comprise higher resolution images with objects typically represented at a larger size or at the extreme as single object shots. In contrast, object density in many of our images is high (~100 objects in some images) and many objects overlap with one another in the field of view.

This means that the task of localizing an object within an image, as measured by mAP, is particularly challenging. Further, as stated above, the number of images in our labelled data is many orders of magnitude smaller than what is typically needed to train an object detection algorithm.

The network that was trained on 750 images achieved a mAP of 0.218 on the validation set of 250 images, crossing our performance threshold for data labelling. We also trained, in distinct trials, on 100, 200, 300, 400, 500 images (stratified the same way as described in the main text) and fitted a curve to model the resultant mAPs as a function of the number of images used for training. We found that mAP followed an approximately logarithmic relationship with the number of images used for training (R^2 = 0.978), as also seen in prior work[3]. With such a gain, a five-fold increase in data labelling beyond the selected number of 1,000 would only yield a +0.07 increase in mAP, guiding our choice of 750 for training optimisation and 1,000 for retraining the final network.

On the independent test set of 250 images, the final model achieved a mAP of 0.211 when averaged across objects, close to a predicted value of 0.23; when weighted by frequency of different objects mAP was 0.318. Individual object categories similar to those found in MS-COCO (people, most types of vehicles, umbrellas and animals) generally achieved higher mAP than those of more novel classes such as market stalls, cooking pots/bowls, debris and trash (Supplementary Table 2). When the threshold for intersection-over-union was fixed at 0.5, the mAP increased for all object categories, more than doubling for categories whose boundaries are harder to identify (e.g., cooking pot/bowl, trash, food and market stall). The model identified zero instances of market vendors, loudspeakers and cookstoves during testing, possibly because they are novel, variable in their appearance and overlap with other categories (e.g., market vendors

were identified as persons, which is correct but incomplete). There were nonetheless exceptions – for example, taxis (which are a type of car) were identified with a mAP of 0.480.

S7. Fixed site data down-sampling for hour of day analysis

When reporting object counts by time of day, we down-sampled the object-counts data from fixed sites so that fixed site data, which recorded images for 15 months, contributed the same amount of data as rotating sites, which recorded images for a single week each. Down-sampling was carried out for images containing each object type, at each hourly interval and at each of the ten fixed sites. For a given hourly interval at a given site, the counts of an object were ordered and every n^{th} image was selected so that the resulting total number of down-sampled images was about 84, equivalent to 2,016 (= 24 × 84) images for an entire week (exactly one week's image quantity at a rotating site). For example, for images containing umbrellas at a site with one camera that took 3,678 images from 00:00:00 to 00:59:59 over its entire 15 months of operation, we ordered the images by the counts of umbrella from smallest to largest and selected every 45th image. Selecting every 45th image gave 82 (from 3,678/45) images which is close to the target of 84 in an hour at a rotating site. In addition to every 45th count, the smallest and largest counts were also selected in order to preserve the full range of the distribution within each hour. Through the ordered sampling, each down-sampled hourly distribution has the same object count distribution as the original hourly-distribution range which is harder to preserve with simple random sampling.

References

1. Liu, J., Zhang, S., Wang, S. & Metaxas, D. Multispectral Deep Neural Networks for Pedestrian Detection. in *Proceedings of the British Machine Vision Conference 2016* 73.1-73.13 (British Machine Vision Association, 2016). doi:10.5244/C.30.73.
2. Peppa, M. V., Bell, D., Komar, T. & Xiao, W. Urban Traffic Flow Analysis Based on Deep Learning Car Detection from CCTV Image Series. *ISPRS - Int. Arch. Photogramm. Remote Sens. Spat. Inf. Sci.* **624**, 499–506 (2018).
3. Sun, C., Shrivastava, A., Singh, S. & Gupta, A. Revisiting Unreasonable Effectiveness of Data in Deep Learning Era. in *2017 IEEE International Conference on Computer Vision (ICCV)* 843–852 (IEEE, 2017). doi:10.1109/ICCV.2017.97.
4. Fortin, F.-A., De Rainville, F.-M., Gardner, M.-A. G., Parizeau, M. & Gagné, C. DEAP: evolutionary algorithms made easy. *J. Mach. Learn. Res.* **13**, 2171–2175 (2012).
5. Huang, J. *et al.* Speed/Accuracy Trade-Offs for Modern Convolutional Object Detectors. in *2017 IEEE Conference on Computer Vision and Pattern Recognition (CVPR)* 3296–3297 (IEEE, 2017). doi:10.1109/CVPR.2017.351.
6. Zoph, B. *et al.* Learning Data Augmentation Strategies for Object Detection. in *Computer Vision – ECCV 2020* (eds. Vedaldi, A., Bischof, H., Brox, T. & Frahm, J.-M.) 566–583 (Springer International Publishing, 2020). doi:10.1007/978-3-030-58583-9_34.
7. Lopes, R. G., Yin, D., Poole, B., Gilmer, J. & Cubuk, E. D. Improving Robustness Without Sacrificing Accuracy with Patch Gaussian Augmentation. *ArXiv190602611 Cs Stat* (2019).
8. Padilla, R., Netto, S. L. & Silva, E. A. B. da. A Survey on Performance Metrics for Object-Detection Algorithms. in *2020 International Conference on Systems, Signals and Image Processing (IWSSIP)* 237–242 (2020). doi:10.1109/IWSSIP48289.2020.9145130.
9. Lin, T.-Y. *et al.* Microsoft COCO: Common Objects in Context. in *Computer Vision – ECCV 2014* (eds. Fleet, D., Pajdla, T., Schiele, B. & Tuytelaars, T.) 740–755 (Springer International Publishing, 2014). doi:10.1007/978-3-319-10602-1_48.

Supplementary Figure 1. Extent of missingness of images.

For each site, the figure shows the proportion of target measurement period with zero, one or two cameras (for sites with two cameras) and zero or one camera (for sites with one camera). Rotating sites are ordered by data availability.

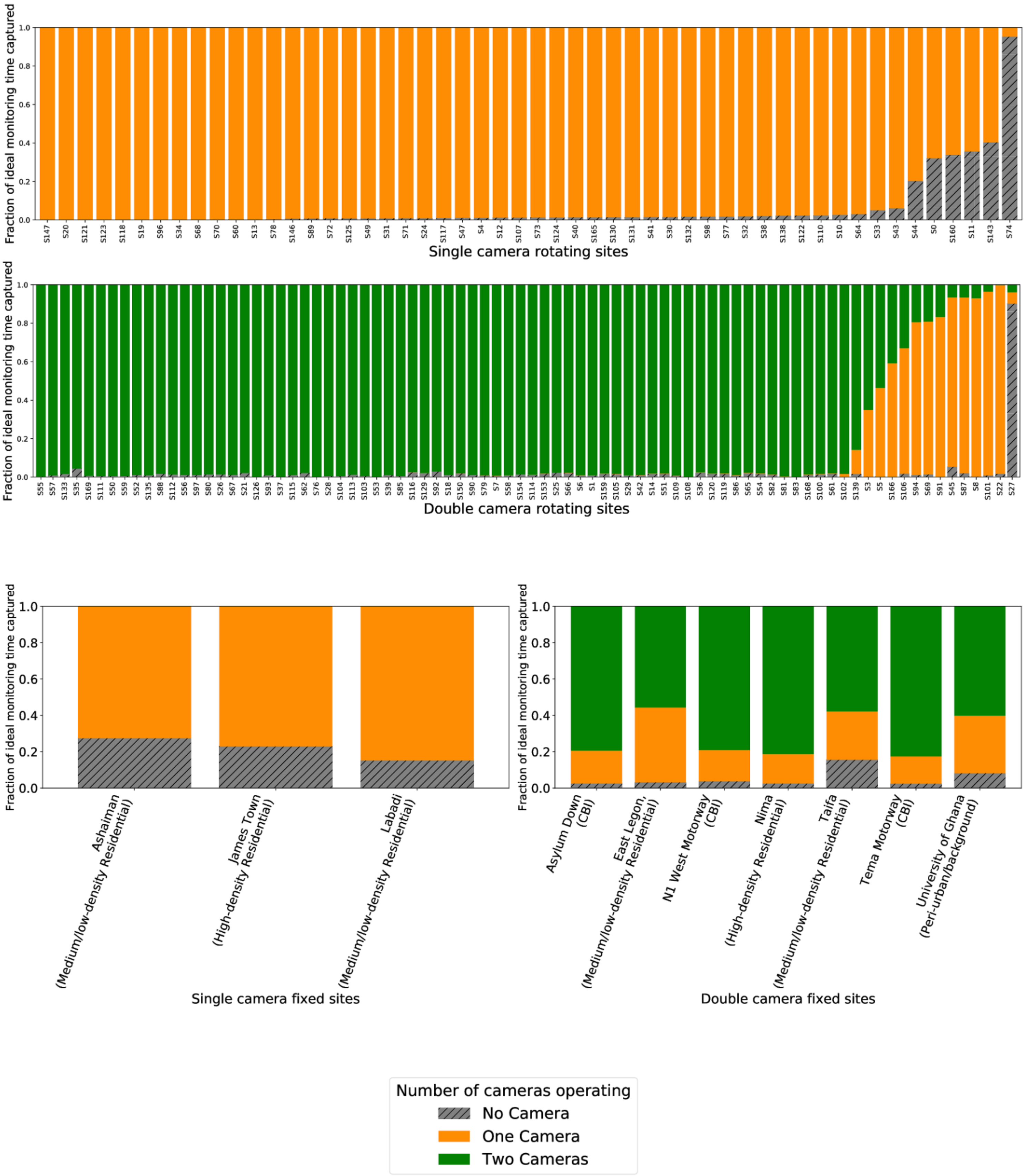

Fraction of ideal monitoring time captured
Single camera rotating sites
S147 S20 S121 S123 S118 S19 S96 S34 S68 S70 S60 S13 S78 S146 S89 S72 S125 S49 S31 S71 S24 S117 S47 S4 S12 S107 S73 S124 S40 S165 S130 S131 S41 S30 S132 S98 S77 S32 S38 S138 S122 S110 S10 S64 S33 S43 S44 S0 S160 S11 S143 S74
Double camera rotating sites
S55 S57 S133 S35 S169 S111 S50 S59 S52 S135 S88 S112 S56 S97 S80 S26 S67 S21 S126 S93 S37 S115 S62 S76 S28 S104 S113 S103 S53 S39 S85 S116 S129 S92 S18 S150 S90 S79 S7 S58 S154 S114 S153 S25 S66 S6 S1 S159 S105 S29 S42 S14 S51 S109 S108 S36 S120 S119 S86 S65 S54 S82 S81 S83 S168 S100 S61 S102 S139 S3 S5 S166 S106 S94 S69 S91 S45 S87 S8 S101 S22 S27
Ashaiman (Medium/low-density Residential)
James Town (High-density Residential)
Labadi (Medium/low-density Residential)
Single camera fixed sites
Asylum Down (CBI)
East Legon, (Medium/low-density Residential)
N1 West Motorway (CBI)
Nima (High-density Residential)
Taifa (Medium/low-density Residential)
Tema Motorway (CBI)
University of Ghana (Peri-urban/background)
Double camera fixed sites
Number of cameras operating
No Camera
One Camera
Two Cameras

Supplementary Figure 2. Distribution of selected object counts across images by day of week.

The distributions are shown for fixed sites because at these sites data were collected over an entire year which means each day of week was sampled multiple times. The figure used data for daytime (06:00-18:00) because night time variability was smaller. The figures show the cumulative probability distribution (CDF) which shows the cumulative percentage of images at each object count. The higher the CDF curve, the smaller the object count, and vice versa.

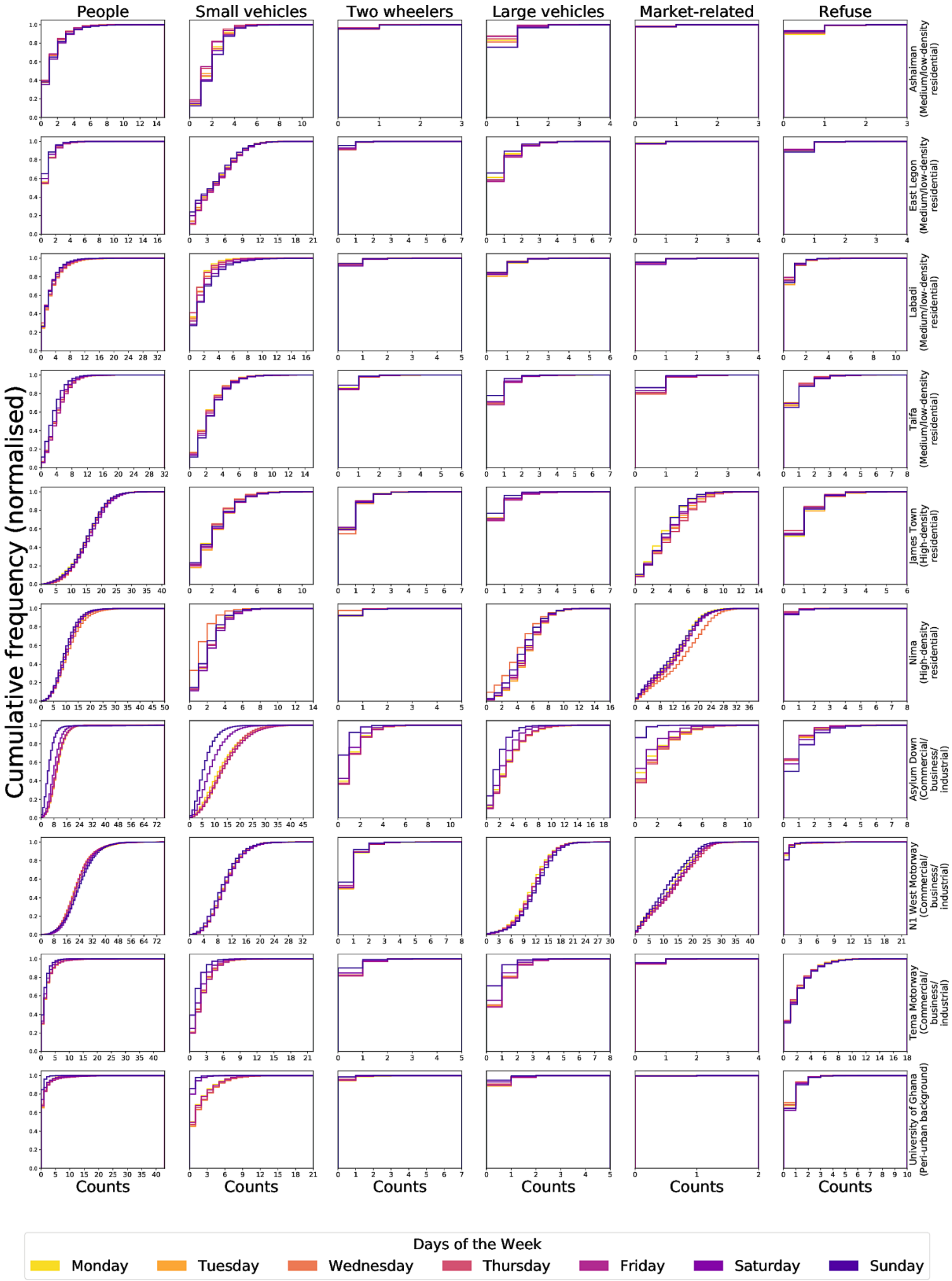

People
Small vehicles
Two wheelers
Large vehicles
Market-related
Refuse
Ashaiman (Medium/low-density residential)
East Legon (Medium/low-density residential)
Labadi (Medium/low-density residential)
Taifa (Medium/low-density residential)
James Town (High-density residential)
Nima (High-density residential)
Asylum Down (Commercial/ business/ industrial)
N1 West Motorway (Commercial/ business/ industrial)
Tema Motorway (Commercial/ business/ industrial)
University of Ghana (Peri-urban background)
Cumulative frequency (normalised)
Counts
Days of the Week
Monday
Tuesday
Wednesday
Thursday
Friday
Saturday
Sunday

Supplementary Figure 3. Co-occurrence of objects in images.

The figure shows the correlation coefficient among object categories, calculated across all images. The number in parentheses shows the p-value for the correlation coefficient.

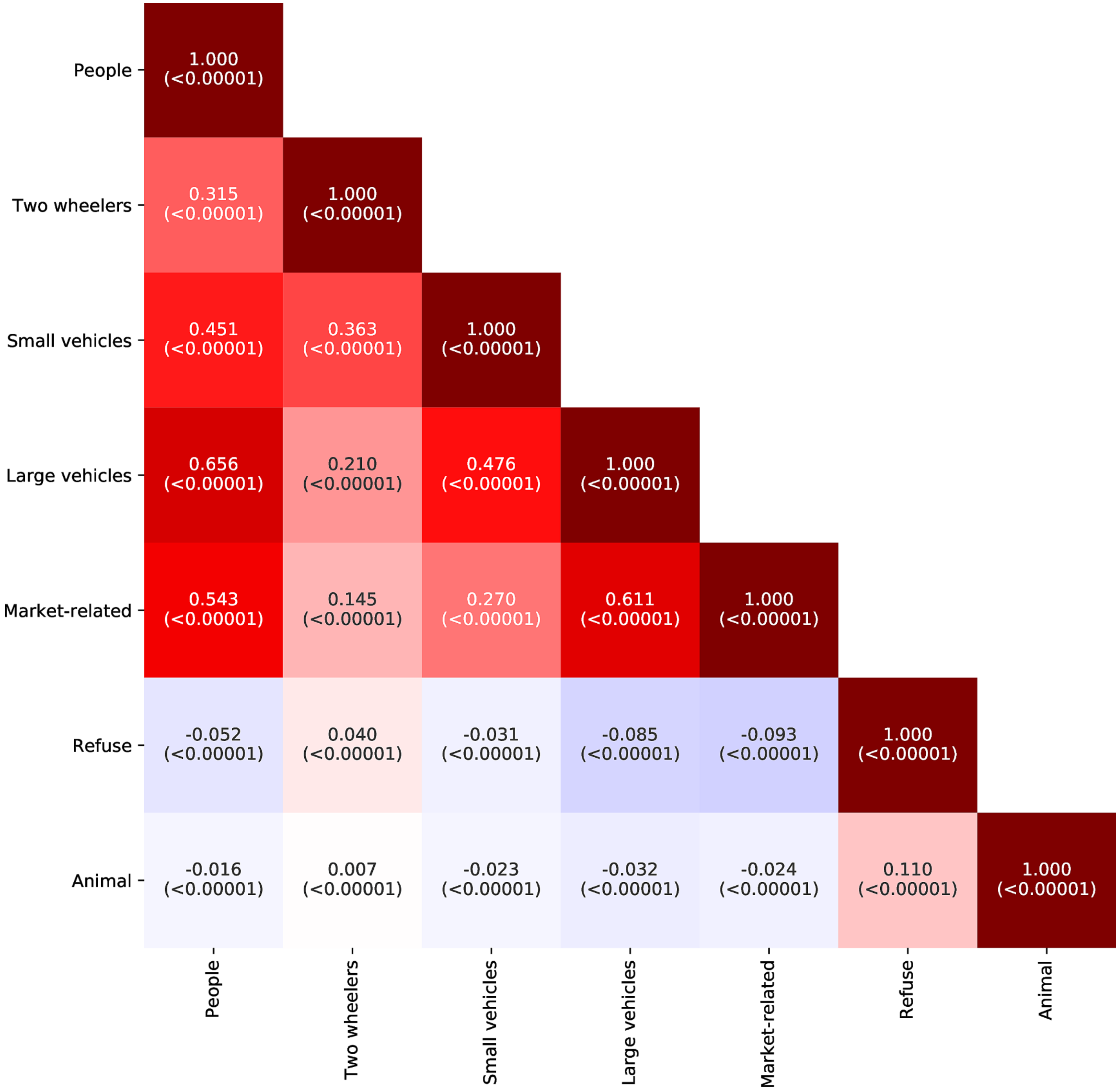
People
Two wheelers
Small vehicles
Large vehicles
Market-related
Refuse
Animal
1.000 (<0.00001)
0.315 (<0.00001)
1.000 (<0.00001)
0.451 (<0.00001)
0.363 (<0.00001)
1.000 (<0.00001)
0.656 (<0.00001)
0.210 (<0.00001)
0.476 (<0.00001)
1.000 (<0.00001)
0.543 (<0.00001)
0.145 (<0.00001)
0.270 (<0.00001)
0.611 (<0.00001)
1.000 (<0.00001)
-0.052 (<0.00001)
0.040 (<0.00001)
-0.031 (<0.00001)
-0.085 (<0.00001)
-0.093 (<0.00001)
1.000 (<0.00001)
-0.016 (<0.00001)
0.007 (<0.00001)
-0.023 (<0.00001)
-0.032 (<0.00001)
-0.024 (<0.00001)
0.110 (<0.00001)
1.000 (<0.00001)
People
Two wheelers
Small vehicles
Large vehicles
Market-related
Refuse
Animal

Supplementary Figure 4. Object count distributions at different hours of the day at fixed sites.

Each panel shows the distribution of object counts at each hour of the day for fixed sites. The sites are divided by land-use type: low- and medium-density (formal) residential; informal, mostly high-density, settlements and slums; commercial, business and industrial areas; and peri-urban areas that are predominantly forest, farmland, grassland or barren land.

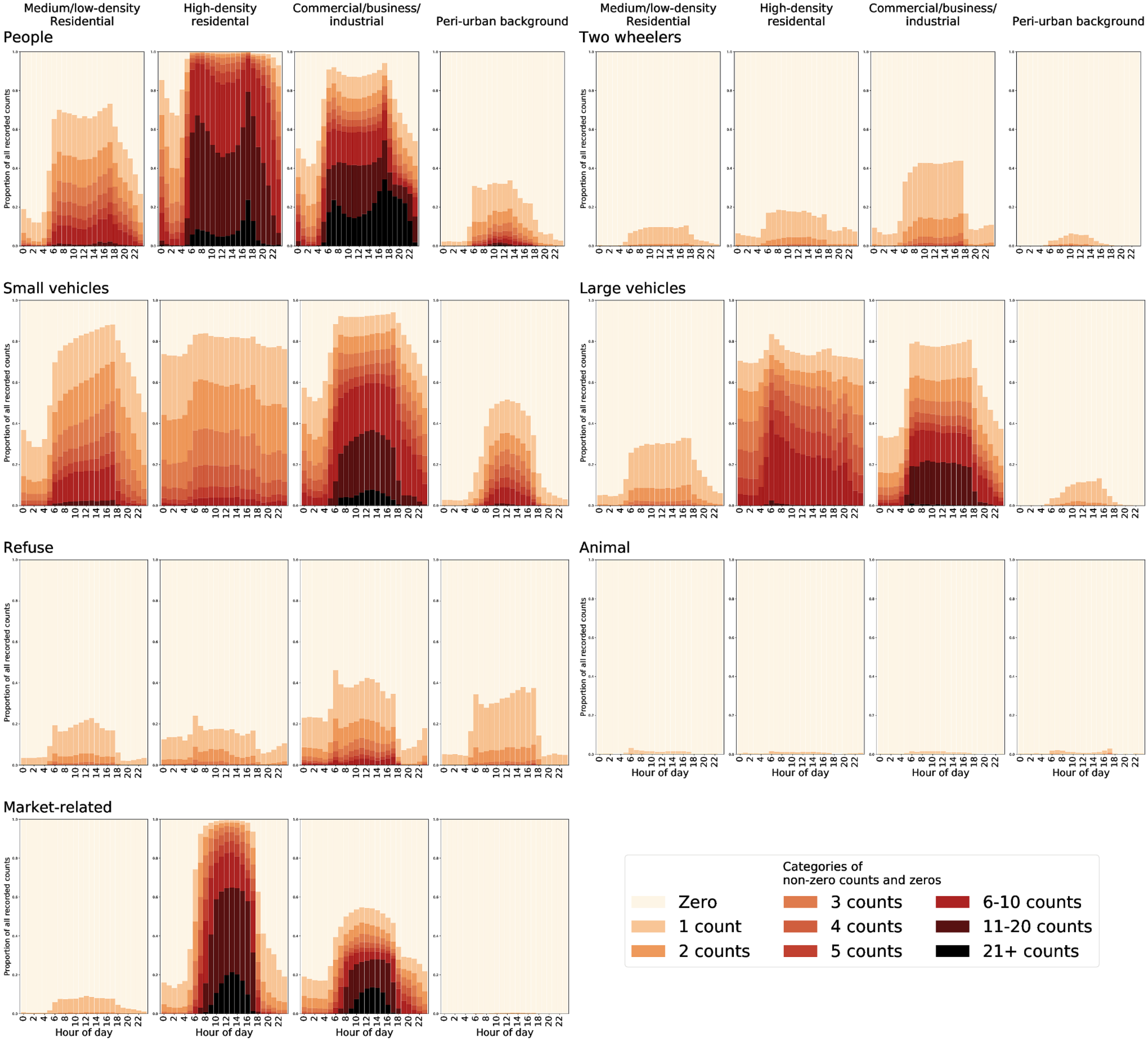

Medium/low-density Residential
High-density residental
Commercial/business/ industrial
Peri-urban background
People
Two wheelers
Small vehicles
Large vehicles
Refuse
Animal
Market-related
Proportion of all recorded counts
Hour of day
Categories of non-zero counts and zeros
Zero
1 count
2 counts
3 counts
4 counts
5 counts
6-10 counts
11-20 counts
21+ counts

Supplementary Figure 5. Object count distributions at different hours of the day at rotating sites.

Each panel shows the distribution of object counts at each hour of the day for rotating sites. The sites are divided by land-use type: low- and medium-density (formal) residential; informal, mostly high-density, settlements and slums; commercial, business and industrial areas; and peri-urban areas that are predominantly forest, farmland, grassland or barren land.

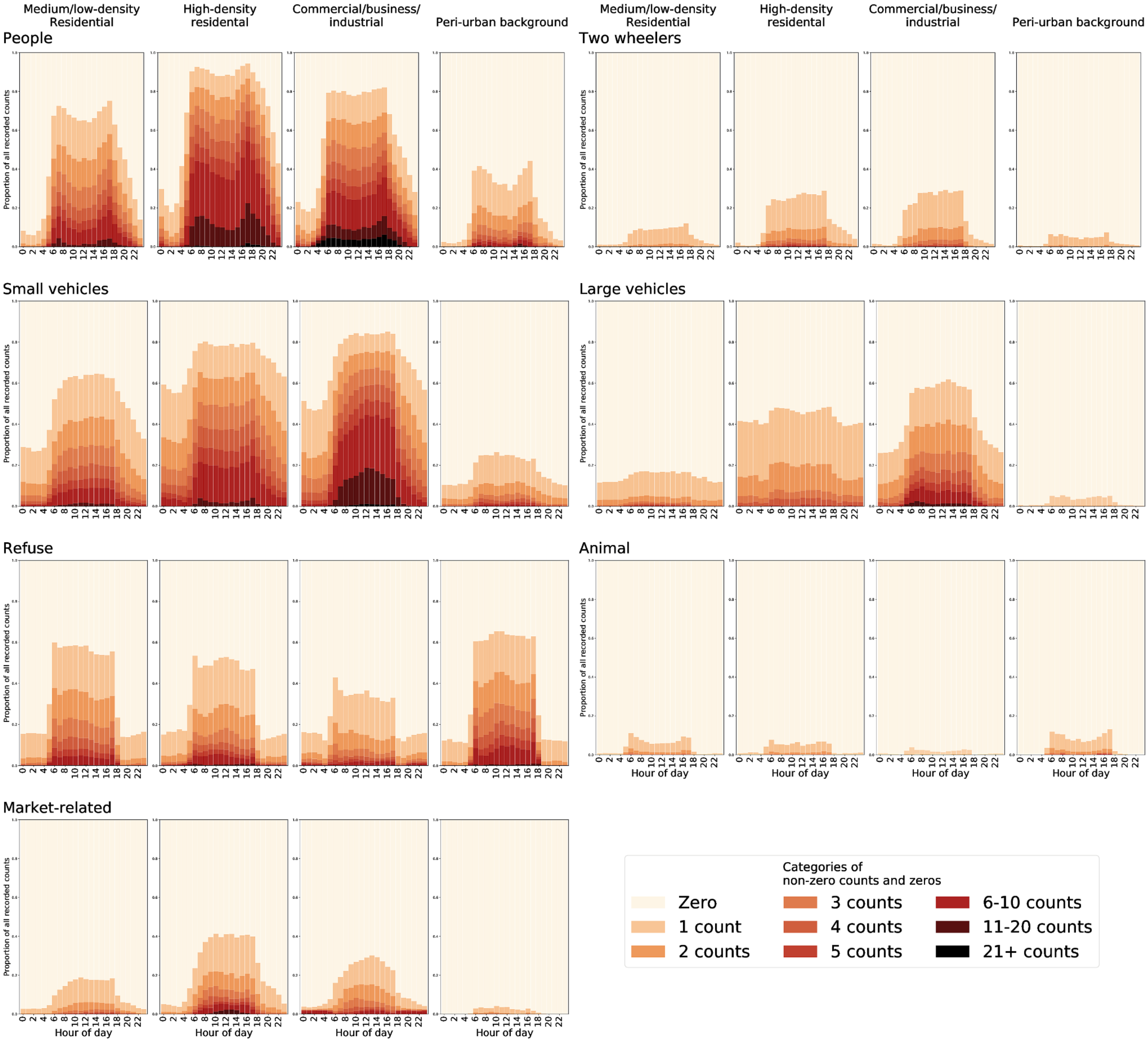

Medium/low-density Residential
High-density residental
Commercial/business/ industrial
Peri-urban background
People
Two wheelers
Small vehicles
Large vehicles
Refuse
Animal
Market-related
Proportion of all recorded counts
Hour of day
Categories of non-zero counts and zeros
Zero
1 count
2 counts
3 counts
4 counts
5 counts
6-10 counts
11-20 counts
21+ counts

Supplementary Table 1. Performance of the retrained model before and after optimisation.

For each object category, and across all categories combined, the table shows mean average performance (mAP), which is defined in SI S6, before and after optimizing the training procedure. The network was trained on the 750 images in the training set and tested on the 250 images in the validation set.

Object	Counts in combined training set (750 images)	Counts in validation set (250 images)	mAP‡ before optimisation	mAP‡ after optimisation	Percentage change
Person	2,543	788	0.408	0.408	0%
Car	981	321	0.364	0.457	+26%
Trash	637	189	0.117	0.122	+4%
Tro tro	437	114	0.416	0.427	+3%
Debris	392	118	0.057	0.057	0%
Umbrella	359	119	0.404	0.445	+10%
Taxi	267	96	0.343	0.464	+35%
Cooking bowl/pot	108	35	0.070	0.144	+106%
Pick-up truck	105	35	0.273	0.249	-9%
Market stall	97	36	0.065	0.152	+134%
Food	99	29	0.121	0.143	+18%
Motorcycle	95	31	0.360	0.345	-4%
Lorry	82	25	0.166	0.244	+47%
Van	79	26	0.069	0.121	+75%
Street vendor	77	20	0.001	0.035	+3500%
Animal	55	19	0.178	0.185	+5%
Bicycle	40	13	0.333	0.384	+15%
Bus	15	5	0.002	0.115	+5750%
Cookstove	11	3	0	0	0%
Loudspeaker	11	2	0	0	0%
Total (average of categories)	6,490	2,024	0.187	0.225	20%

‡ Mean average precision mAP was calculated as described in SI S6 with varying intersection-over-union thresholds

Supplementary Table 2. Performance of the final object detection model.

For each object category, and across all categories combined, the table shows mean average performance (mAP), which is defined in SI S6. The network was trained on the 1,000 images in the training and validation sets and tested on the 250 images in the test set.

Object	Counts in combined training and validation set (1,000 images)	Counts in testing set (250 images)	mAP‡	mAP@0.5‡
Person	3,331	855	0.389	0.728
Car	1,302	336	0.400	0.673
Trash	826	211	0.092	0.205
Tro tro	551	139	0.367	0.622
Debris	510	128	0.061	0.122
Umbrella	478	139	0.437	0.735
Taxi	363	85	0.480	0.681
Cooking bowl/pot	143	34	0.072	0.181
Pick-up truck	140	34	0.272	0.425
Market stall	133	35	0.098	0.192
Food	128	32	0.064	0.165
Motorcycle	126	31	0.314	0.729
Lorry	107	28	0.336	0.473
Van	105	26	0.044	0.088
Street vendor	97	22	0	0
Animal	74	19	0.295	0.579
Bicycle	53	13	0.195	0.375
Bus	20	5	0.307	0.438
Cookstove	14	4	0	0
Loudspeaker	13	4	0	0
Total (frequency weighted)	8,514	2180	0.318	0.575
Total (average of categories)	NA	NA	0.211	0.370

‡ Mean average precision mAP was calculated as described in SI S6 with varying intersection-over-union thresholds, and mAP@0.5 with a single intersection-over-union threshold of 0.5.
